# Antifungal Compounds from Cyanobacteria

**DOI:** 10.3390/md13042124

**Published:** 2015-04-13

**Authors:** Tânia K. Shishido, Anu Humisto, Jouni Jokela, Liwei Liu, Matti Wahlsten, Anisha Tamrakar, David P. Fewer, Perttu Permi, Ana P. D. Andreote, Marli F. Fiore, Kaarina Sivonen

**Affiliations:** 1 Department of Food and Environmental Sciences, Viikki Biocenter 1, University of Helsinki, FI-00014, Helsinki, Finland; E-Mails: tania.shishido@helsinki.fi (T.K.S.); anu.humisto@helsinki.fi (A.H.); jouni.jokela@helsinki.fi (J.J.); liwei.liu@helsinki.fi (L.L.); matti.wahlsten@helsinki.fi (M.W.); anisha.tamrakar@helsinki.fi (A.T.); david.fewer@helsinki.fi (D.P.F.); 2 Program in Structural Biology and Biophysics, Institute of Biotechnology/Nuclear Magnetic Resonance Laboratory, University of Helsinki, FI-00014, Helsinki, Finland; E-Mail: perttu.permi@helsinki.fi; 3 Center for Nuclear Energy in Agriculture, University of São Paulo, Avenida Centenário 303, Piracicaba, 13400-970, São Paulo, Brazil; E-Mails: apdini@cena.usp.br (A.P.D.A.); fiore@cena.usp.br (M.F.F.)

**Keywords:** natural products, drug leads, *Candida albicans*, *Aspergillus* sp., 16S rRNA

## Abstract

Cyanobacteria are photosynthetic prokaryotes found in a range of environments. They are infamous for the production of toxins, as well as bioactive compounds, which exhibit anticancer, antimicrobial and protease inhibition activities. Cyanobacteria produce a broad range of antifungals belonging to structural classes, such as peptides, polyketides and alkaloids. Here, we tested cyanobacteria from a wide variety of environments for antifungal activity. The potent antifungal macrolide scytophycin was detected in *Anabaena* sp. HAN21/1, *Anabaena cf. cylindrica* PH133, *Nostoc* sp. HAN11/1 and *Scytonema* sp. HAN3/2. To our knowledge, this is the first description of *Anabaena* strains that produce scytophycins. We detected antifungal glycolipopeptide hassallidin production in *Anabaena* spp. BIR JV1 and HAN7/1 and in *Nostoc* spp. 6sf Calc and CENA 219. These strains were isolated from brackish and freshwater samples collected in Brazil, the Czech Republic and Finland. In addition, three cyanobacterial strains, *Fischerella* sp. CENA 298, *Scytonema hofmanni* PCC 7110 and *Nostoc* sp. N107.3, produced unidentified antifungal compounds that warrant further characterization. Interestingly, all of the strains shown to produce antifungal compounds in this study belong to Nostocales or Stigonematales cyanobacterial orders.

## 1. Introduction

Cyanobacteria are photosynthetic cosmopolitan prokaryotic organisms that have been isolated from aquatic (freshwater, brackish and marine), terrestrial (soil, lichen-associated and the surface of leaves), and different aquatic and terrestrial extreme environments (hot springs, high salinity, deserts) [1,2]. In these environments, cyanobacteria face competitors and predators, including parasitic fungi, such as chytrids. The production of oligopeptides by *Planktothrix* spp. is believed to contribute to the defense against chytrid fungi [3,4]. Antifungal compounds have been previously detected in cyanobacterial extracts, such as fischerellin A, hapalindole, hassallidin/balticidins, carazostatin, phytoalexin, tolytoxin, scytophycin, toyocamycin, tjipanazole, nostocyclamide, nostodione and nostofungicidine [5,6,7]. Most of these compounds are synthetized by ribosomal pathways or by nonribosomal pathways. Examples of enzymes involved in the nonribosomal pathways are nonribosomal peptide synthetase (NRPS), polyketides synthases (PKS) or hybrid systems of both NRPS/PKS. The NRPSs and PKSs are multifunction modular enzymes involved in the synthesis of nonribosomal peptides and polyketides [8,9]. In cyanobacteria, the antifungal hassallidin is synthesized by NRPSs and tailoring enzymes [10]. Interestingly, a single hassallidin gene cluster encoded in the biosynthetic pathway for more than 40 chemical variants of hassallidin in *Anabaena* sp. SYKE748A [10].

Invasive fungal infections caused by *Candida* spp. and *Aspergillus* spp. are common, especially in immunocompromised patients. The increase of antifungal resistance indicates an urgent need for new antifungal compounds [11,12,13]. Here, we screened cyanobacteria for antifungal compounds, and we were able to detect known, but also potential new antifungal natural products.

## 2. Results and Discussion

### 2.1. Cyanobacteria Producing Antifungal Compounds

We screened 194 cyanobacterial strains isolated from brackish water, freshwater and terrestrial habitats using a disc diffusion assay (Table 1, Supplementary Table S1). Freeze-dried cells of cyanobacteria were extracted with methanol and were tested by the disc diffusion assay against *Candida albicans* and/or *Aspergillus flavus*. Ten strains were found to produce bioactive compounds inhibiting *Candida albicans* and nine inhibiting *Aspergillus flavus* (Table 1, Supplementary Figure S1). These eleven strains with antifungal compounds were analyzed using LC-MS.

**Table 1:** Cyanobacterial strains producing antifungal compounds according to the disc diffusion assay.

Cyanobacteria	16S rRNA Gene	Origin	Inhibited Organism(s)	Antifungal Compound
*Nostoc* sp. CENA 219	KP701037	Benthic freshwater, Brazil	*Ca*/*Af*	hassallidin
*Anabaena* sp. BIR JV1	KP701036	The Gulf of Finland	*Ca*	hassallidin
*Anabaena* sp. HAN7/1	KP701033	Epilithic, Finland	*Ca*	hassallidin
*Nostoc calcicula* 6 sf Calc	KP701034	Dobre Pole, Czech Republic	*Ca*/*Af*	hassallidin
*Anabaena* sp. HAN21/1	KP701032	Gastropod, Finland	*Ca*/*Af*	scytophycin
*Anabaena cf. cylindrica* PH133	AJ293110	Lake Arresø, Denmark	*Ca*/*Af*	scytophycin
*Scytonema* sp. HAN3/2	KP701039	Green biofilm in the pond, Finland	*Ca*/*Af*	scytophycin
*Nostoc* sp. HAN11/1	KP701035	Small pond on a rock, Finland	*Ca*/*Af*	scytophycin
*Fischerella* sp. CENA 298	KP701038	Soil, Brazil	*Ca*/*Af*	unidentified
*Scytonema hofmanni* PCC 7110	NR112180	Limestone, Bermuda	*Ca*/*Af*	unidentified
*Nostoc* sp. N107.3	KP701040	Lichen, Finland	*Af*	unidentified

*Ca* = *Candida albicans*; *Af* = *Aspergillus flavus*.

**Figure 1:**
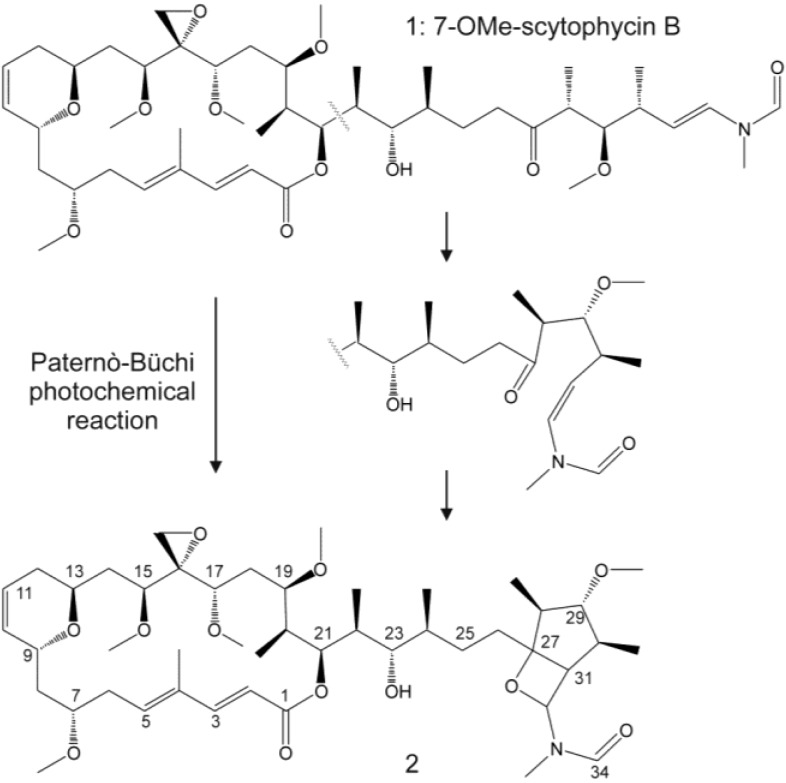
7-OMe-scytophycin-B (**1**) isolated from *Anabaena* sp. HAN21/1 and 7-OMe-scytophycin-B reacted by light (**2**) after the Paternò–Büchi reaction. Stereochemistry is according to the literature.

Antifungal compounds in *Anabaena* sp. HAN21/1, *Anabaena cf*. *cylindrica* PH133, *Nostoc* sp. HAN11/1 and *Scytonema* sp. HAN3/2 were identified to be scytophycins (Sc) with LC-MS. The identification based on the molecular masses of the compounds showed losses of multiple 32-Da neutral fragments (typical for aliphatic methoxy groups containing compounds). In addition, the ^15^N-labeling experiments of *Anabaena* sp. HAN21/1 showed that these compounds contained a single nitrogen atom (Supplementary Figure S2). To confirm the identity of these compounds, the main antifungal compound of *Anabaena* sp. HAN21/1 was isolated by HPLC. NMR analysis (Supplementary Figures S3 and S4 and Table S2) showed that this compound was 7-OMe-Sc-B (**1**, Figure 1). The NMR sample contained also another compound scytophycin analog **2**, the structure of which can be derived from 7-OMe-Sc-B via the photocatalytic Paternò–Büchi reaction (Figure 1). This 7-OMe-Sc-B preparation presented a MIC/IC_50_ of 0.33/0.16 mg·mL^−1^ (0.40/0.19 mM) for *Candida albicans* HAMBI 484 and 0.67/0.29 mg·mL^−1^ (0.80/0.23 mM) for *Candida guilliermondii* HAMBI 257.

**Figure 2:**
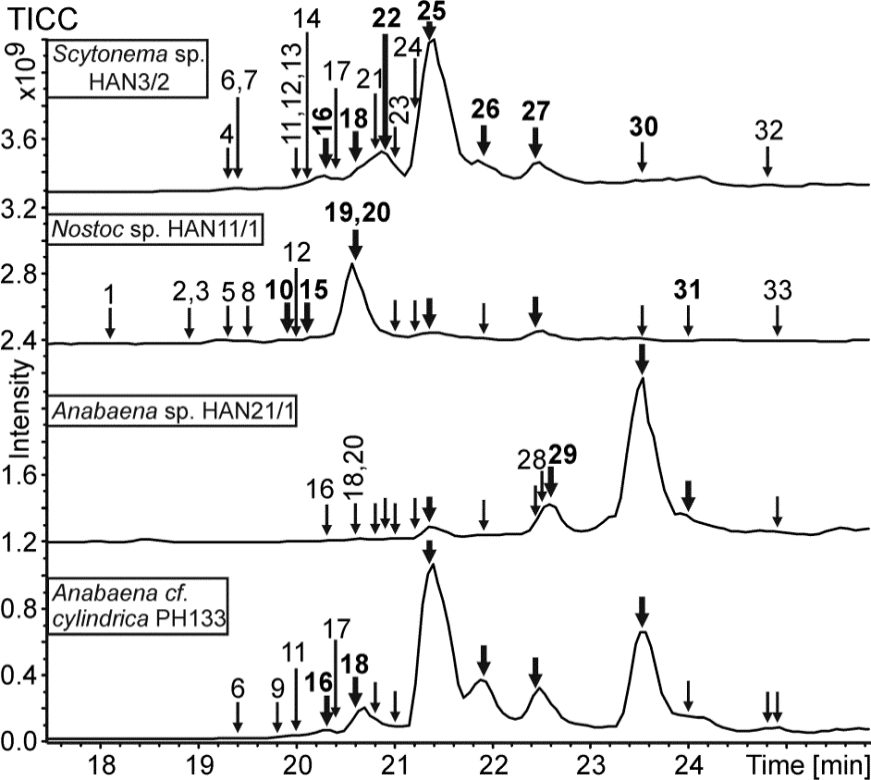
Total ion current chromatograms of methanol extracts of *Scytonema* sp. HAN3/2, *Nostoc* sp. HAN11/1, *Anabaena* sp. HAN21/1 and *Anabaena cf. cylindrica* PH133 showing the elution of scytophycin variants 1–33. Bolded numbers and arrows show the main scytophycin variants.

Several scytophycin variants were identified from strains *Anabaena* sp. HAN21/1, *Anabaena cf*. *cylindrica* PH133, *Nostoc* sp. HAN11/1 and *Scytonema* sp. HAN3/2 (Figure 2, Table 2 and Supplementary Table S3). These strains produced known and previously unreported scytophycins variants (Table 2 and Supplementary Table S3 and S4), and their identification was based on the existence of prominent [M + Na]^+^ and also often [M + H − H_2_O]^+^ ions with masses at the range of reported scytophycins (Supplementary Figure S2) and of the characteristic product ion spectra of [M + Na]^+^ (example spectrum from 7-OMe-Sc-B in Supplementary Figure S5). Detailed mass spectrometric structural analysis turned out to be challenging. Protonated dehydrated scytophycins generated product ion spectra full of ions for which many represent the loss of water and methanol (32 Da), and hence, they were of small value for the determination of the structure (Supplementary Figure S6). Nitrogen labeling confirmed that the even number product ions contained the nitrogen atom of the scytophycins (Supplementary Figures S6 and S7). Product ion spectra from sodiated scytophycins were more structurally informative, showing fewer product ions, as seen from the spectrum of sodiated 7-OMe-Sc-B (Supplementary Figure S5). Nitrogen labeling again confirmed the presence of nitrogen in the product ions (Supplementary Figure S7). The base peaks in the scytophycin product ion spectra (for example, *m*/*z* 620 from 7-OMe-Sc-B) and ion *m*/*z* 262 contained nitrogen (Supplementary Figure S7). Based on the aforementioned data, the tentative fragmentation and structure of 33 scytophycins are presented in Supplementary Tables S3 and S5.

**Table 2:** Scytophycin variants, retention times (R_t_), experimental (Exp) and calculated (Cal) [M + Na]^+^ ion masses (*m/z*), mass error in ppm (**∆**), formula and abundance (+, ++, +++), in the studied *Scytonema* sp. HAN3/2, *Nostoc* sp. HAN11/1, *Anabaena* sp. HAN21/1 and *Anabaena cf. cylindrica* PH133 strains. ^a^ Mass from [M + H − H_2_O]^+^, because [M + Na]^+^ was absent. New variants are indicated by “N” and in bold.

							Strains			
Scytophycin (Sc) variant		R_t (min)_	[M + Na]^+^ (*m/z*)				HAN3/2	HAN11/1	HAN21/1	PH133
No.	Chemical variant		Exp	Cal	∆ (ppm)	Formula				
**10 N**	**6-OH-7-OMe-15-O-deMe-Sc-B**	**19.9**	**858.5**					++		
15	6-OH-7-OMe-Sc-D/E	20.1	874.5					++		
**16 N**	**X-OH-Sc-D/E (not 6-OH)**	**20.3**	**860.5116**	**860.5131**	**−1.70**	**C_45_H_75_NO_13_**	++		+	++
18	Sc-D/E	20.6	844.5157	844.5181	−2.90	C_45_H_75_NO_12_	++		+	++
**19 N**	**Sc**	**20.6**	**814.5^a^**					++		
20	6-OH-7-OMe-Sc-B	20.6	872.5					+++	+	
**22 N**	**Sc**	**20.9**	**842.5**				++		+	
25	Sc-B	21.3	842.5022	842.5025	−0.35	C_45_H_73_NO_12_	+++	++	++	+++
**26 N**	**Sc, (-O from C15/16/17/19)**	**21.9**	**826.5062**	**826.5076**	**−1.67**	**C_45_H_73_NO_11_**	++	+	+	++
27	Sc-C	22.4	828.5221	828.5232	−1.37	C_45_H_75_NO_11_	++	++	+	++
**29 N**	**7-OMe-29-OAc-Sc-B**	**22.6**	**884.5129**	**884.5131**	**−0.18**	**C_47_H_75_NO_13_**			++	
30	7-OMe-Sc-B (1)	23.5	856.5				+	+	+++	+++
**31 N**	**7-OMe-Sc, (-O from C15/16/17)**	**24.0**	**840.5**					+	++	+

**Figure 3:**
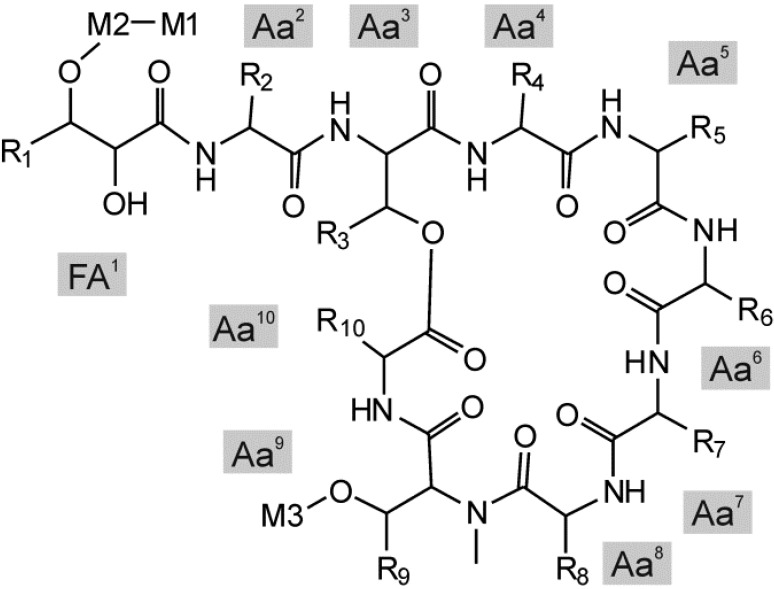
The general chemical structure of hassallidin. M_1_–M_3_ are monosaccharides, R_1_ is the hydrocarbon chain of the fatty acid chain FA^1^ and R_2_–R_10_ side chains of amino acids Aa^2^ to Aa^10^.

**Figure 4:**
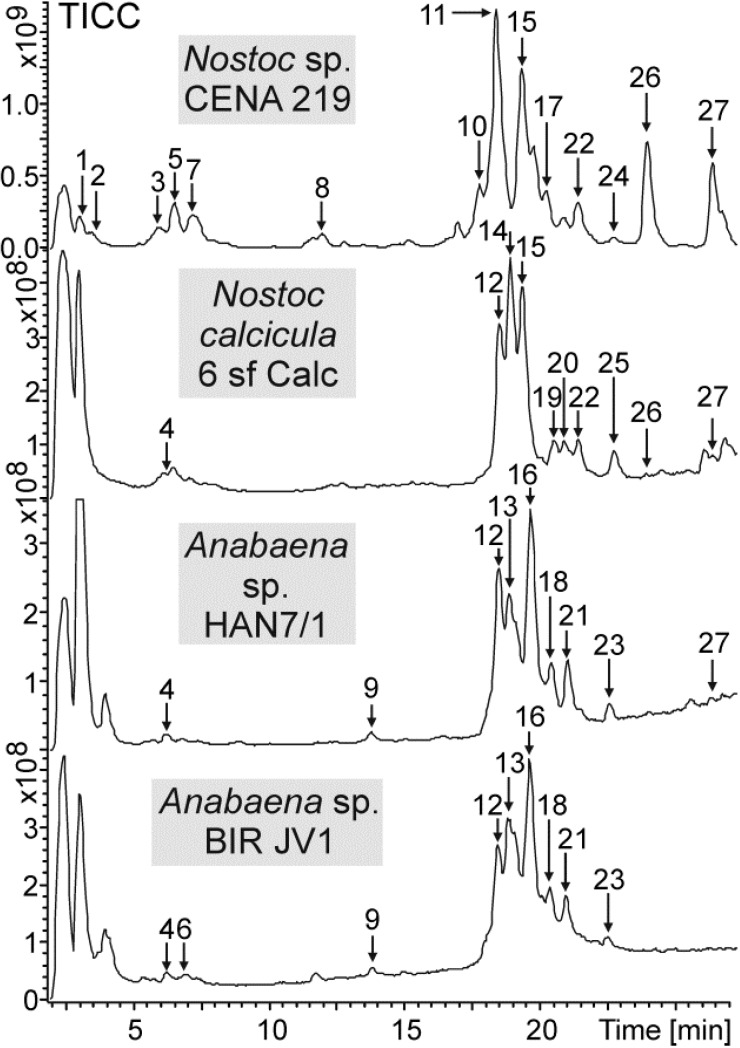
Total ion current chromatograms of methanol extracts of *Nostoc* sp. CENA 219, *Nostoc calcicula* 6 sf Calc, *Anabaena* sp. HAN7/1 and *Anabaena* sp. BIR JV1 showing the elution of hassallidin variants 1–27.

Fourteen variants of hassallidin (Figure 3) were detected in the *Nostoc* sp. CENA 219 methanol extract (Figure 4, Table 3). The hassallidin variants differ in the presence of sugars (pentose, deoxyhexose, hexose, acetylated hexose and N-acetylhexosamine), by the composition of the aglyconic peptide core structure and the fatty acid moiety (Table 3). The relative amounts of hassallidin variants can be roughly estimated from the TIC chromatograms presented in Figure 4. Variants 11, 15 and 26 are the most abundant hassallidins in *Nostoc* sp. CENA 219. They all have the same aglyconic lipopeptide structure *m/z* 1298 but the number of monosaccharides differs. *N*-acetylhexosamine was found only from the Brazilian *Nostoc* sp. CENA 219. Ten variants of hassallidins were detected in the *Nostoc calcicula* 6 sf Calc, nine variants from the *Anabaena* sp. BIR JV1 and *Anabaena* sp. HAN7/1 methanol extract (Figure 4, Table 3 and Supplementary Figures S9–S15). The main hassallidin variants found in the extract of *Nostoc calcicula* 6 sf Calc were 12, 14 and 15. The hassallidin 12 was also detected in the *Anabaena* spp. BIR JV1 and HAN7/1 and the hassallidin 15 in the *Nostoc* sp. CENA 219 cells extracts. Variants 12, 13 and 16 were the most abundant hassallidins in *Anabaena* sp. BIR JV1 and *Anabaena* sp. HAN7/1, respectively. They have the same aglyconic lipopeptide structure *m/z* 1,298 but the degree of acetylation on hexose in the position M3 varies (Figure 3). Altogether, hassallidin profiles of *Anabaena* sp. BIR JV1 and *Anabaena* sp. HAN7/1 were highly similar. The structure of the aglyconic lipopeptide *m/z* 1298 is unknown. The hassallidin structures have been deduced from the mass spectra presented in the Supplementary Figures S9–S15.

**Table 3:** Retention times (R_t_), ion masses and monosaccharide (M1, M2 and M3) content of hassallidin variants in *Nostoc* sp. CENA 219, *Nostoc calcicula* 6 sf Calc, *Anabaena* sp. HAN7/1 and *Anabaena* sp. BIR JV1 strains. The major variants produced are highlighted in grey.

							Strains			
	R_t_	Ion masses (*m*/*z*)		Monosaccharides			CENA 219	6sf Calc	HAN7/1	BIR JV1
No	(min)	AL [M + H]^+^	[M + Na]^+^	M1	M2	M3				
1	3.1	1296.6	1815.8	HexNAc	Pent	Hex	x			
2	3.5	1294.8	1813.7	HexNAc	Pent	Hex	x			
3	5.9	1280.7	1799.8	HexNAc	Pent	Hex	x			
4	6.2	1280.7	1772.8	Hex	dHex	Hex		x	x	x
5	6.5	1278.6	1797.8	HexNAc	Pent	Hex	x			
6	6.8	**1280.7**	1814.9	Hex	dHex	AcHex				x
7	7.2	1314.7	1833.7	HexNAc	Pent	Hex	x			
8	11.9	1270.7	1789.7	HexNAc	Pent	Hex	x			
9	13.7	1236.7	1728.8	Hex	dHex	Hex			x	x
10	17.7	1282.7	**1801.9**	HexNAc	Pent	Hex	x			
11	18.3	1298.7	1817.8	HexNAc	Pent	Hex	x			
12	18.4	1298.7	1790.6	Hex	dHex	Hex		x	x	x
13	18.8	1298.7	1832.8	Hex	dHex	AcHex			x	x
14	18.9	1298.7	**1790.8**	Hex	dHex	Hex		x		
15	19.3	1298.6	**1614.7**		Pent	Hex	x	x		
16	19.6	1298.7	1874.8	Hex	dHex	diAcHex			x	x
17	20.2	1264.7	**1783.9**	HexNAc	Pent	Hex	x			
18	20.3	1264.7	1756.6	Hex	dHex	Hex			x	x
19	20.5	1298.7	**1628.7**		dHex	Hex		x		
20	20.9	1264.7	**1756.8**	Hex	dHex	Hex		x		
21	21.0	1298.7	1916.8	Hex	dHex	triAcHex			x	x
22	21.4	1264.7	**1580.8**		Pent	Hex	x	x		
23	22.5	1280.6	1958.7	Hex	dHex	tetraAcHex			x	x
24	22.7	1266.7	1785.8	HexNAc	Pent	Hex	x			
25	22.7	1298.7	**1452.7**		Pent			x		
26	23.9	1298.7	1482.7			Hex	x	x		
27	26.3	1264.7	1448.7			Hex	x	x	x	

AL = Aglyconic lipopeptide containing substructures FA^1^ and Aa^2^–Aa^10^, HexNAc = N-acetylhexosamine (residue mass 203 Da), Pent = pentose (132), dHex = deoxyhexose (146), Hex = hexose (162), tetra/tri/di/AcHex = tetra-, tri-, di- or monoacetylhexose (330, 288, 246, 204).

The methanol extract of *Scytonema hofmanni* PCC 7110 was fractionated by HPLC and tested against *Aspergillus flavus*. However, the bioactivity was lost during the re-isolation of the fraction containing the antifungal compound.

### 2.2. Evolutionary Relation of the Cyanobacterial Strains Producing Antifungal Compounds

**Figure 5:**
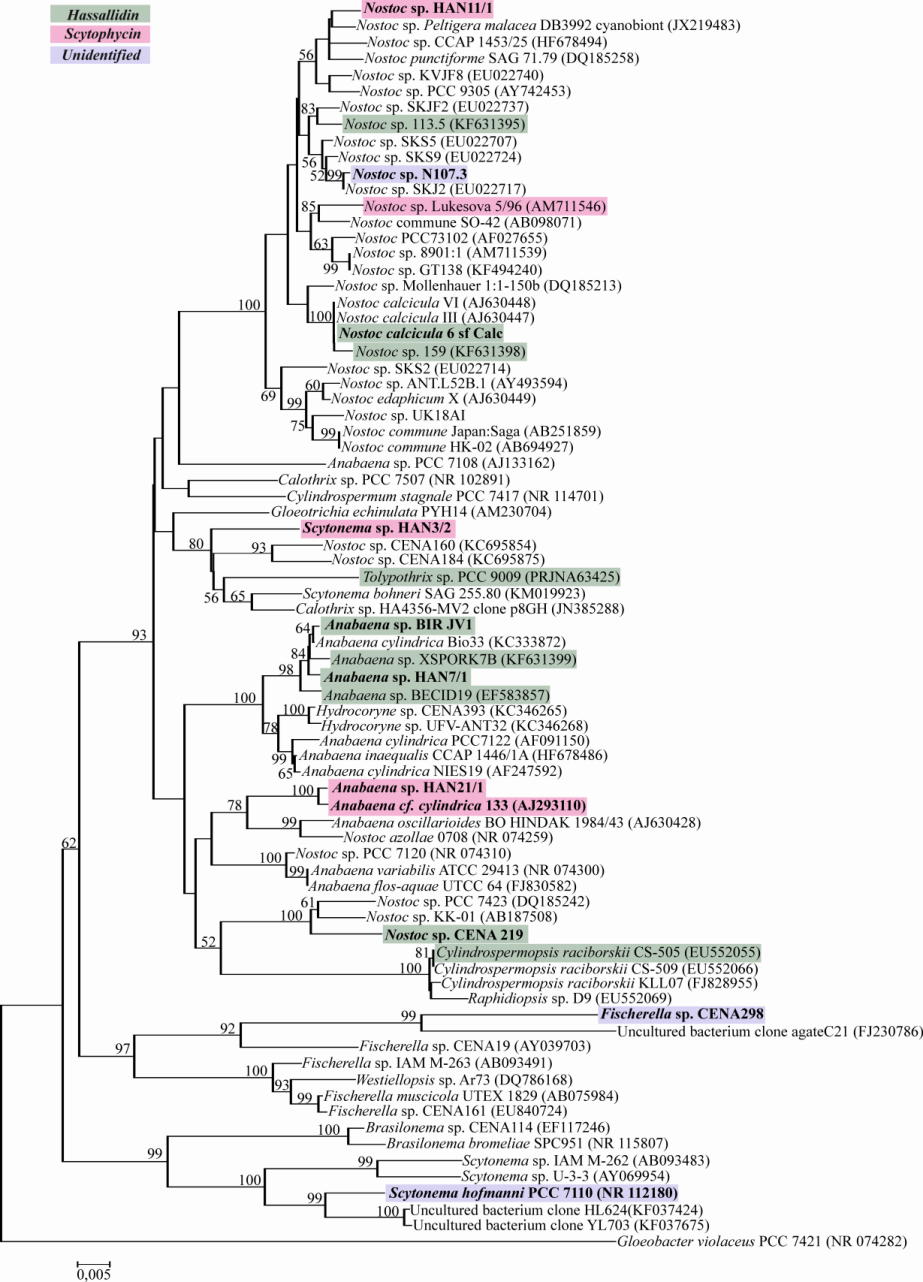
The distribution of cyanobacterial strains producing antifungal compounds. The neighbor-joining phylogenetic tree is based on the 16S rRNA genes sequences constructed with 1000 bootstraps in which the percentages over 50 are indicated in each node. Studied strains are in bold.

A phylogenetic tree was constructed based on partial 16S rRNA gene sequences of the cyanobacteria producing antifungal compounds and other strains retrieved from the NCBI database (Figure 5). Analyzing the *Anabaena* sp. HAN21/1 16S rRNA gene sequence similarity of 99% with *Anabaena cf. cylindrica* PH133 in GenBank (NCBI) compelled us to check if this strain could produce scytophycin. Interestingly, through the phylogenetic analysis, we found another *Anabaena* strain isolated from a different environment producing scytophycins.

### 2.3. Discussion

The screening for antifungal compounds produced by cyanobacteria led us to discover new strains producing scytophycins and hassallidins. Antifungal compounds were detected from strains belonging to the Nostocales and Stigonematales orders, such as *Anabaena*, *Fischerella*, *Nostoc* and *Scytonema*. Phylogenetic analysis based on 16S rRNA gene sequences shows that the studied strains are widely distributed in the tree (Figure 5). It is common to have non-producers and producers of bioactive compounds grouped together in phylogenetic trees [14,15]. We first detected *Anabaena* sp. HAN21/1, isolated from a gastropod living in brackish water in the coast of Finland, producing scytophycins. The comparison of the 16S rRNA gene sequence of this strain with other sequences in the NCBI led us to investigate if the first hit, *Anabaena cf. cylindrica* PH133, could also produce scytophycin. Surprisingly, we detected *Anabaena cf. cylindrica* PH133, isolated from freshwater of Lake Arresø in Denmark producing scytophycins. Interestingly, this is the first report to our knowledge of *Anabaena* strains producing scytophycins. Two other benthic cyanobacteria from the Baltic Sea coast, *Nostoc* sp. HAN11/1 and *Scytonema* sp. HAN3/2, were also detected to produce scytophycin (Table 1 and Table 2).

The studied strains produced a diverse variety of scytophycin, including scytophycin B, C, D/E (Table 2). Scytophycins have been previously reported to be produced by *Scytonema* spp. [16,17], *Cylindrospermum* [18] and *Nostoc* sp. 5/96 [19]. Tolytoxin, which was first detected in *Tolypothrix conglutinata* var. *colorata*, is also known as 6-hydroxy-7-*O*-methyl-scytophycin B [16,17]. Fourteen scytophycin variants have been described by other authors before, in which the six variable positions reported are C-6, -7, -16, -19, -23 and -27 (Supplementary Table S4). The main chemical variant isolated from *Anabaena* sp. HAN21/1 was 7-OMe-scytophycin-B, which had been previously reported from *Nostoc* sp. 5/96 [19]. Nitrogen labeling of *Anabaena* sp. HAN21/1 confirmed the presence of nitrogen in the product ions. The base peaks in the scytophycin product ion spectra (for example, *m*/*z* 620 from 7-OMe-Sc-B) and ion *m*/*z* 262 contained nitrogen (Supplementary Figure S7). However, the product ions structures for ion *m*/*z* 262 and *m*/*z* 620 reported in Tomsickova *et al.*, 2013, did not contain nitrogen, which is missing from the scytophycin structures that they reported. The *Scytonema*, *Tolypothrix*, *Cylindrospermum* and *Nostoc* strains described to produce scytophycins were all isolated from terrestrial habitats [18,19].

Scytophycins are macrolides polyketides, but the biosynthetic genes involved in their synthesis are unknown. Other compounds that have been described to be structurally related to scytophycins are lobophorolide [20], swinholides, aplyronine, sphinxolides/reidispongiolides and ulapualides/kabiramide/halichondramides/mycalolides [21]. Aplyronine A, sphinxolide, mycalolide A and ulapualide A have very similar side chains to scytophycins, with all presenting an *N*-methylvinylformamide [21]. Interestingly, these compounds have been found in other organism than cyanobacteria: seaweed *Lobophora variegate*, a symbiont in the red sea sponge *Theonella swinhoei*, sea hare *Aplysia kurodai*, an unidentified nudibranch, marine sponges *Neosiphonia superstes* and *Reidispongia coerulea*, egg masses of the nudibranch *Hexabranchus sanguineas*, sponges *Halichondria* sp. and *Mycale* spp. and hard coral *Tubastraea faulkneri* [21]. Swinholide A, scytophycins, sphinxolide, ulapualides, mycolides and aplyronine A have potent cytotoxicity against cancer cell lines [21]. Interestingly, tolytoxin has been proposed to play an ecological role in the defense system of *Scytonema ocellatum* [22]. The cyanobacteria increased the synthesis of tolytoxin when in the presence of the fungal cell-wall polysaccharide chitin [22].

We report for the first time to our knowledge a variant of the scytophycin isolated from *Anabaena* sp. HAN21/1, which can be altered after a Paternò–Büchi reaction catalyzed by light (Figure 1). This photochemical reaction modifies the side chain and probably was responsible for the loss of activity of 7-OMe-scytophycin-B.

Hassallidins are lipopeptides produced by nonribosomal biosynthetic enzymes in cyanobacteria [10,23,24]. Hassallidins have been detected previously in *Cylindrospermopsis*, *Anabaena*, *Aphanizomenon*, *Nostoc*, *Tolypothrix* and *Hassalia* [10,23,24]. In this study, diverse variants of hassallidins were detected in methanol extracts from strains *Nostoc* spp. 6 sf Calc and CENA 219 and *Anabaena* spp. BIR JV1 and HAN7/1 (Table 3).

The *Nostoc* sp. CENA 219 hassallidins’ (Table 3) monosaccharide composition was similar to *Nostoc* sp. 113.5 monosaccharides [10], but the aglyconic lipopeptide structures were more variable in the present studied strains. *Nostoc calcicula* 6 sf Calc hassallidin structures were similar to *Nostoc* sp. 159 and *Tolypothrix* sp. PCC 9009 hassallidin structures (Supplementary Material in [10]). *Anabaena* sp. BIR JV1 and *Anabaena* sp. HAN7/1 hassallidins (Table 3) monosaccharide composition was similar to *Anabaena* spp. XPORK 5C, XSPORK 7B and BECID 19 monosaccharides [10], but the aglyconic lipopeptide structures were more variable in the strains in the present study. The detailed aglyconic lipopeptide structure is difficult to analyze reliably from the product ion spectrum of the aglyconic lipopeptide ion and could be determined by NMR analysis of purified hassallidin variants. Alternatively, the study of the substrate specificities of the adenylation domains of the hassallidin genes N, O, V and Y could be used to predict which amino acids are incorporated into the peptide [10].

Balticidins belongs to the family of hassallidin compounds, and they have been recently detected in *Anabaena cylindrica* Bio33 [25]. Balticidins are cyclic or linear peptides that differ from hassallidin by the presence of arabinose and galacturonic acid and the possible presence of chlorine in the fatty acid chain [25]. Hassallidins and balticidins are known for their activity against *Candida* spp. [10,25,26]. Hassallidins A and B were found to be active against *Cryptococcus neoformans*, *Aspergillus* spp., *Fusarium* spp., *Penicillium* sp., *Ustilago maydis* and *Acremonium strictum* [26]. In addition, hassallidins A and B were active against human acute T-cell leukemia (Jurkat ATCC-TIB-152) and murine aneuploid fibrosarcoma (L 929) [26]. Hassallidin D was shown to be a more potent antifungal than hassallidin A and B [10]. However, several variants of hassallidins are produced in trace amounts by the cyanobacteria, and the heterologous expression or syntheses of these variants is necessary to evaluate the activity of these compounds. The high amount of variants of hassallidins discovered recently highlights the potential of cyanobacteria for the synthesis of antifungal compounds. There is a need for further studies to analyze the potential of these new variants as antifungal and anticancer drugs.

Our screening study also revealed cyanobacterial strains producing unidentified antifungal compounds. *Fischerella* sp. CENA 298, *Scytonema hofmanni* PCC 7110 and *Nostoc* sp. N107.3 presented antifungal activity. LC-MS analysis of methanol crude extracts and isolated compounds by HPLC did not reveal insights into the chemical structure of these antifungal compounds. Further analyses are necessary for isolation and characterization of these bioactive compounds.

## 3. Experimental Section

### 3.1. Cultivation of Cyanobacterial Strains

The cyanobacterial strains were cultivated in Z8 medium [27] with or without a nitrogen source, in BG-11 [28] with, without or containing half of the amount of nitrogen, AA (Allen and Arnon) medium [29] or ASM medium [30]. The strains were cultivated in the specific medium (40–3000 mL) at 17 °C–25 °C under continuous light of 3–15 μmol·m^−2^·s^−1^. The strain *Anabaena* sp. HAN21/1 was labeled with ^15^N and analyzed with LC/MS, as previously described [31].

### 3.2. Extraction of Intracellular Cyanobacterial Compounds

The cells analyzed were obtained either from a 40-mL culture centrifuged at 8000× *g* for 5 min or from freeze-dried biomass (up to 100 mg). The fresh culture cells from the 40 mL of medium were extracted with 1 mL of methanol and glass beads (0.5-mm diameter glass beads, Scientific Industries INC) using a FastPrep cell disrupter instrument three times for 30 s at a speed of 6.5 m·s^−1^. The samples were centrifuged for 10,000× *g* for 5 min at room temperature and kept in a glass tube. The cells were extracted a second time using another 1 mL of methanol, as described before, and both methanol extracts were combined and dried with a stream of air. Residues were re-suspended in 200 μL of methanol, sonicated (Sonorex super 10P, Bandelin, Berlin, Germany) and kept at −20 °C for further analysis. A total of 100 mg of the freeze-dried biomass were extracted two times with 1 mL of methanol, as described before. The obtained supernatant was reserved for further analysis.

### 3.3. Disc Diffusion Assay

Methanol extracts (100–400 μL) from all of the studied strains were applied to a paper disc (Abtek Biologicals Ltd., Liverpool, United Kingdom). *Candida albicans* HAMBI261 was grown in yeast and mold agar (YM) medium, while *Candida albicans* HAMBI484 and *Aspergillus flavus* HAMBI 829 were grown in potato dextrose agar (PDA) medium. The inoculum for the bioassay was prepared as previously described [10]. The discs containing crude extract were applied on the PDA or YM media plates containing *Candida albicans* HAMBI 261 or HAMBI 484 and/or *Aspergillus flavus* HAMBI 829. After a period of 24 h for yeast at 35 °C or 48 h at 28 °C for mold, the plates were analyzed for inhibition zones, which were measured including the paper disk diameter.

### 3.4. Chemical Analysis

The obtained methanol extracts that contained antifungal activity were studied by LC-MS (Agilent 1100 Series LC/MSD Trap XCT Plus, Agilent Technologies, Palo Alto, CA, USA). All samples were analyzed with the following protocol: Luna C_8_(2) column (150 × 4.60 mm, 5 μm, 100 Å, Phenomenex, Torrance, CA, USA) with two eluents (A: 0.1% HCOOH in water, Fluka, Sigma-Aldrich (St. Louis, MO, USA); and B: 2-propanol in 0.1% HCOOH, Optima^®^ LC/MS quality, Fischer Scientific, Fair Lawn, NJ, USA), used in a linear gradient, where Eluent B increased from 5% to 100% in 35 min with flow rate of 0.15 mL·min^−1^ and an injection volume of 10 μL. The mass spectrometer trap drive value was 144.0 with a scan range of *m*/*z* 200–1100. Samples suggesting signs of hassallidins were re-analyzed with the following protocol: Luna C_18_(2) column with 0.1% HCOOH in water or acetonitrile was used in the linear gradient, where the concentration of the acetonitrile eluent was increased from 30% to 70% in 49 min with a flow rate of 0.15 mL·min^−1^ and an injection volume of 10 μL. The mass spectrometer trap drive value was 110 with a scan range of *m*/*z* 300–2200. These obtained UV-, MS- and MS^2^-product ion spectrums were analyzed.

### 3.5. Purification of Scytophycin

HPLC (HP 1100 Series, Agilent, with DAD) was used to isolate the main scytophycin variant from the methanol extract of strain *Anabaena* sp. HAN21/1. The methanol extract was evaporated to dryness, and the residue was dissolved in HPLC eluent (40% isopropanol, 60% 0.1% HCOOH; 700 μL of eluent to every 1 mL of methanol extract), which was then injected in 100-μL batches into the Luna C_8_(2) column (150 × 4.60 mm, 5 μm, 100 Å, Phenomenex, Torrance, CA, USA). The column was eluted isocratically with a flow rate of 0.75 mL·min^−1^. Peaks eluting at 13.5 min were collected and combined in one tube. The eluent was evaporated, and the residue was dissolved in methanol, which was applied to the SP column (Phenomenex Strata C18-E 55 μm, 70 Å, 5 g/20 mL Giga Tubes, Torrance, CA USA), eluted with methanol. Dried and [D_6_] DMSO-dissolved compound was analyzed with NMR.

### 3.6. NMR of Scytophycin

The NMR spectra of 7-OMe-scytophycin-B derivative (Figure 1, compound 2) were recorded on the Varian Inova 600 spectrometer equipped with a cryogenically-cooled ^1^H/^13^C/^15^N triple resonance probe head and an actively-shielded z-axis gradient system in [D_6_] DMSO at 301 K. The ^1^H spectrum was measured with 8 transients using 24,000 complex points, which corresponds to an acquisition time of 2 s. Two-dimensional homonuclear total correlation spectroscopy (TOCSY) and double-quantum filtered correlation spectroscopy (DQF-COSY) experiments were both measured with 8 transients per FID (free induction decay), using 300 and 2386 complex points in the t_1_ and t_2_ domains, which translates to acquisition times of 43 and 341 ms in the t_1_ and t_2_ domains. The ^13^C-^1^H heteronuclear single quantum coherence (HSQC) spectrum was acquired with 180 and 596 complex points in t_1_ (^13^C) and t_2_ (^1^H), using 16 transients per FID, corresponding to acquisition times of 6 and 85 ms in the ^13^C and ^1^H dimensions, respectively. The ^13^C heteronuclear multiple bond correlation (^13^C-HMBC) experiment was measured using 256 and 2380 complex points in t_1_ (^13^C) and t_2_ (^1^H). This corresponds to acquisition times of 7.1 and 340 ms in the ^13^C and ^1^H dimensions, respectively. The signals were accumulated with 128 transients. The long-range ^1^H-^13^C delay was set to optimize transfer for 6 Hz ^1^H-^13^C couplings.

### 3.7. Microdilution Assay of Scytophycin

The microdilution assay was performed against *Candida albicans* HAMBI484 and *Candida guilliermondii* HAMBI 257, as previously described [10]. The calculation for the minimum inhibitory concentration (MIC) and half maximum inhibitory concentration (IC_50_) is explained in detail in [10].

### 3.8. DNA Extraction, Sequencing and Phylogenetic Analysis

The DNA extraction of the strains containing antifungal compounds was obtained using the DNeasy Plant Mini Kit (Qiagen Gmbh, Hilden, Germany) or the E.Z.N.A SP Plant DNA Mini Kit (Omega Bio-Tek Inc., Norcross, GA, USA). The cells were homogenized in the FastPrep cell disrupter for 30 s at 6.5 ms^−1^ (repeated three times), then the DNA extraction was followed as described in the manufacturer’s instructions. The partial 16S rRNA gene was amplified by PCR, as previously described [15]. The fragments were cloned into a pCR^®^2.1-TOPO^®^ vector (Invitrogen, Carslbad, CA, USA) and transformed to One Shot^®^ Chemically Competent *Escherichia coli* TOP10 cells, as recommended by the manufacturer. Positive clones were found through PCR using the vector primers (M13F and M13R) and sequenced. The phylogenetic tree using the neighbor-joining method was obtained in MEGA 5.0 [32]. The Kimura 2-parameter model with 1000 bootstrap replications and gamma distributed rates among sites has been used to obtain the phylogenetic tree.

## 4. Conclusions

Cyanobacterial strains were detected producing the antifungal compounds scytophycins and hassallidins. In this study, we first described to our knowledge *Anabaena* spp. producing scytophycins. There is a huge potential for the discovery of new variants of known antifungal compounds (scytophycins and hassallidins) but also for potential new compounds produced by *Fischerella* sp. CENA 298, *Scytonema hofmanni* PCC 7110 and *Nostoc* sp. N107.3 which need to be further analyzed.

## Supplementary Files

Supplementary File 1
