# Supplementary material for: Antifungal Compounds from Cyanobacteria
Source: Mar Drugs. 2015 Apr 13;13(4):2124–40. doi: 10.3390/md13042124 (PMC4413203; doi:10.3390/md13042124)
Supplement: Supplementary File 1 [file marinedrugs-13-02124-s001.pdf]

Supplementary Information

Contents of Tables and Figures

Tables

Table S1. Strains used to screen for antifungal compounds using disc diffusion assay.
Table S2. ^{1}H and ^{13}C NMR data for modified 7-OMe-Scytophycin B (**2**, conformers A and B), unknown 7-OMe-Scytophycin B (C) and 7-OMe-Scytophycin B (**1**, conformers D and E) in [D6] DMSO. ( ) = weak signal, [ ] = split signal.
Table S3. Scytophycin variants (No and Name), retention times (R_t), experimental (Exp) and calculated (Cal) ion masses (*m*/*z*) of $[M + H - H_2O]^+$, $[M + Na]^+$ and $[h - 4H + Na]^+$, mass error (**Δ**) in ppm, molecular formula and frequency in strains HAN3/2 = *Scytonema* sp. HAN3/2, HAN11/1 = *Nostoc* sp. HAN11/1, HAN21/1 = *Anabaena* sp. HAN21/1 and PH133 = *Anabaena cf. cylindrica* PH133. hk = product ion of the sodiated scytophycin, see Figure 2. Accurate mass measurements obtained with Q-TOF and others with ion trap.
Table S4. Previously published scytophycin variants, their monoisotopic masses (M), positions of the variable functional groups. 6-OH-7-OMe-scytophycin B = tolytoxin A. Carbon numbers in Figure 1.
Table S5. Sodiated ion $[M + Na]^+$ mass (*m/z*), product ions and their *m/z*, intensities and comments concerning the tentative structures of scytophycin variants No 1–33. Ion structures a–n are presented in Figure S8.

Figures

Figure S1. Disc diffusion bioassay using cyanobacterial extracts against *Candida albicans* (A) and *Aspergillus flavus* (B). PH133 = *Anabaena cf. cylindrica* PH133; HAN3/2 = *Scytonema* sp. HAN3/2; HAN21/1 = *Anabaena* sp. HAN21/1; HAN11/1= *Nostoc* sp. HAN11/1; HAN7/1 = *Anabaena* sp. HAN7/1; JV1 = *Anabaena* sp. BIR JV1; 219 = *Nostoc* sp. CENA 219; 6sf = *Nostoc calcicula* 6 sf Calc; 298 = *Fischerella* sp. CENA 298; N107 = *Nostoc* sp. N107.3; 7110 = *Scytonema hofmanni* PCC 7110; − is negative control with methanol and + is 0.1mg of nystatin (Sigma-Aldrich, St. Louis, MO, USA).
Figure S2. Nonlabeled (^{14}N) and ^{15}N-labeled sodiated and dehydrated protonated scytophycin ions from *Anabaena* sp. HAN21/1.
Figure S3. NMR spectra of scytophycin preparate containing mainly 7-OMe-scytophycin-B derivative (**2**, Figure 1) and as a minor component native 7-OMe-scytophycin-B **(1,** Figure 1). ^{1}H NMR spectrum (A). Annotated ^{1}H-^{1}H COSY spectra (B and C). ^{1}H-^{1}H TOCSY spectrum (D). Annotated ^{13}C-HSQC spectrum (E). Annotated ^{13}C HMBC spectrum (F, G and H). ○ = signal intensity too low to be seen in ^{13}C-HSQC spectrum.
Figure S4. Structure of photoreacted 7-OMe-scytophycin B (**2**, Figure 1) with ^{1}H-^{1}H DQF-COSY (bold lines) and ^{13}C HMBC (arrows) correlations.
Figure S5. Product ion spectrum from sodiated 7-OMe-scytophycin-B (*m/z* 856.5) produced by *Anabaena* sp. HAN21/1.
Figure S6. Product ion spectrum from dehydrated protonated 7-OMe-scytophycin-B from *Anabaena* sp. HAN21/1.

Figure S7. Nonlabeled (^{14}N) and ^{15}N-labeled product ion spectrum from sodiated 7-OMe-scytophycin-B (**1**, Figure 1 and No 30, Table 2) of *Anabaena* sp. HAN21/1.

Figure S8. Structure of scytophycin-B (No 25 in Tables 2, S2 and S4) showing the product ions (a, b, c, dk, ek, fk, gk, hk, ik, jk, lhk, l, mhk, m and n) studied from the sodiated ion $[M + Na]^+$.

Figure S9. Mass spectra of *Nostoc* sp. CENA 219 hassallidins 1, 2, 3, 5 and 7.

Figure S10. Mass spectra of *Nostoc* sp. CENA 219 hassallidins 8, 11, 15, 22, 26 and 27.

Figure S11. Mass spectra of *Nostoc* sp. CENA 219 hassallidins 10, 17 and 24.

Figure S12. Mass spectra of *Nostoc* sp. 6 sf Calc hassallidins 12, 14, 19 and 25.

Figure S13. Mass spectra of *Anabaena* sp. HAN7/1 hassallidins 18, 20, 21 and 23.

Figure S14. Mass spectra of *Anabaena cylindrica* BIR JV1 hassallidins 4, 6, and 9.

Figure S15. Mass spectra of *Anabaena cylindrica* BIR JV1 hassallidins 13, 16, and 21.

Table S1. Strains used to screen for antifungal compounds using disc diffusion assay.

Genera	Growth Media [#]	Strain	City/State or State	place of Sampling	Year of Isolation	Identification Based on 16S rRNA Sequences
Strains from Brazil						
Nostoc sp.*	AA/Z8x	CENA219	Morro Branco/CE	water accumulated	2009	+
Phormidium sp.	BG50/Z8	CENA270	Paulista/PA	water in the pond	2009	JQ771627
Fischerella sp.*	ASM	CENA298	Paraíba	soil	2009	
Cyanospira *	AA	CENA215	Morro Branco/CE	water accumulated	2009	+
Limnothrix	BG50	CENA217	Morro Branco/CE	water accumulated	2009	
Nostoc *	AA	CENA221	Morro Branco/CE	water accumulated	2009	
Synechococcus	BG50	CENA222	Morro Branco/CE	water accumulated	2009	
Aphanocapsa	BG50	CENA223	Morro Branco/CE	water accumulated	2009	
Geitlerinema	BG50	CENA224	Morro Branco/CE	water accumulated	2009	
Nostoc *	AA	CENA227	Açude São Mateus/CE	water used by population (lake)	2009	
Geitlerinema	BG50	CENA228	Açude São Mateus/CE	water used by population (lake)	2009	
Leptolyngbya	BG50	CENA229	Açude São Mateus/CE	water used by population (lake)	2009	
Synechococcus	BG50	CENA232	Açude São Mateus/CE	water used by population (lake)	2009	
Synechococcus	BG50	CENA233	Açude São Mateus/CE	water used by population (lake)	2009	
Nostoc *	AA	CENA234	Açude São Mateus/CE	water used by population (lake)	2009	
Pseudanabaena	BG50	CENA235	Povoado Novo Aurora/CE	water in the pond	2009	
Planktothrix	BG50	CENA236	Povoado Novo Aurora/CE	water in the pond	2009	
Nostoc *	AA	CENA237	Lagoa Povoado Nova Aurora/CE	water pond used by cattle	2009	
Nostoc *	AA	CENA238	Lagoa Povoado Nova Aurora/CE	water pond used by cattle	2009	
Nostoc *	AA	CENA239	Lagoa Povoado Nova Aurora/CE	water pond used by cattle	2009	
Myxosarcina	BG50	CENA240	Cisterna/Canindé/CE	water in the cistern	2009	
Myxosarcina	BG50	CENA241	Cisterna/Canindé/CE	water in the cistern	2009	+
Myxosarcina	BG50	CENA242	Cisterna/Canindé/CE	water in the cistern	2009	
Myxosarcina	BG50	CENA244	Cisterna/Canindé/CE	water in the cistern	2009	
Anabaena *	BG0	CENA247	Rio Camarão/CE	river water	2009	

Table S1. *Cont.*

Synechococcus	BG50	CENA249	Rio Camarão/CE	river water	2009	
Gloeotrichia *	AA	CENA250	Rio Camarão/CE	river water	2009	
Microchaete *	BG50	CENA251	Rio Camarão/CE	river water	2009	
Geitlerinema	BG50	CENA252	Sodrelândia/BA	water in the pond	2009	
Synechococcus	BG50	CENA253	Sodrelândia/BA	water in the pond	2009	
Leptolyngbya	BG50	CENA254	Sodrelândia/BA	water in the pond	2009	
Leptolyngbya	BG50	CENA256	Sodrelândia/BA	water in the pond	2009	
Nostoc *	AA	CENA257	Sodrelândia/BA	water in the pond	2009	
Nostoc *	AA	CENA258	Sodrelândia/BA	water in the pond	2009	
Nostoc *	BG50	CENA259	Sodrelândia/BA	water in the pond	2009	
Nostoc *	AA	CENA261	Sodrelândia/BA	water in the pond	2009	
Chroococcales	BG0	CENA263	Baixão dos Bois/BA	water accumulated	2009	
Merismopedia	BG50	CENA264	Icó/CE	water accumulated	2009	+
Pseudanabaena	ASM	CENA266	Paulista/PA	water in the pond	2009	
Pseudanabaena	BG50	CENA267	Paulista/PA	water in the pond	2009	
Scytonema *	BG50	CENA268	Paulista/PA	water in the pond	2009	
Nostoc *	ASM	CENA269	Paulista/PA	water in the pond	2009	
Nostoc *	ASM	CENA271	Paulista/PA	water in the pond	2009	
Gloeotrichia *	BG50	CENA272	Paulista/PA	water in the pond	2009	
Nostoc *	ASM	CENA274	Bahia	soil	2009	
Nostoc *	ASM	CENA275	Bahia	soil	2009	
Chroococcales	AA	CENA276	Bahia	soil	2009	
Nostoc *	AA	CENA277	Bahia	soil	2009	
Nostoc *	BG0	CENA278	Bahia	soil	2009	
Lyngbya	BG50	CENA281	Piauí	soil	2009	
Leptolyngbya	ASM	CENA282	Piauí	soil	2009	

Table S1. *Cont.*

Calothrix *	ASM	CENA283	Piauí	soil	2009	
Gloeotrichia *	ASM	CENA285	Piauí	soil	2009	
Pseudanabaena	BG11	CENA287	Piauí	soil	2009	
Gloeotrichia *	AA	CENA288	Ceará	soil	2009	
Stigonema *	BG50	CENA291	Ceará	soil	2009	
Leptolyngbya	BG50	CENA292	Ceará	soil	2009	
Nostoc *	BG50	CENA293	Ceará	soil	2009	
Nostocales *	ASM	CENA294	Ceará	soil	2009	
Stigonema *	BG50	CENA295	Ceará	soil	2009	
Nostoc *	ASM	CENA296	Ceará	soil	2009	
Leptolyngbya	BG11	CENA299	Rio Grande do Norte	soil	2009	
Strains from Finland						
Unknown *	Z8x	HAN3/1	Kobben, Hanko	green biofilm in the pond	2012	
Scytonema sp.*	Z8x	HAN3/2	Kobben, Hanko	green biofilm in the pond	2012	
Unknown *	Z8x	HAN6/1	Kobben, Hanko	Growth on rock waterline	2012	
Calothrix sp.*	Z8x	HAN6/3	Kobben, Hanko	Growth on rock waterline	2012	+
Calothrix sp. *	Z8x	HAN6/4	Kobben, Hanko	Growth on rock waterline	2012	+
Unknown *	Z8x	HAN7/1	Kobben, Hanko	small stones in the water	2012	
Calothrix sp. *	Z8x	HAN8/2	Kobben, Hanko	Growth on rock waterline	2012	+
Nostoc sp. *	Z8x	HAN10/1	Kobben, Hanko	small pond on a rock	2012	+
Nostoc sp. *	Z8x	HAN11/1	Kobben, Hanko	small pond on a rock	2012	+
Calothrix sp. *	Z8x	HAN12/2	Kobben, Hanko	black spots film, benthic	2012	+
Anabaena sp. *	Z8x	HAN15/2	Kobben, Hanko	Gastropod from waterline	2012	+
Unknown *	Z8x	HAN15/3	Kobben, Hanko	Gastropod from waterline	2012	
Calothrix sp. *	Z8x	HAN16/2	Kobben, Hanko	Waterplant from shallow water	2012	+

Table S1. *Cont.*

Calothrix sp. *	Z8x	HAN17/1	Kobben, Hanko	Red biofilm	2012	+
Calothrix sp. *	Z8x	HAN19/2	Kobben, Hanko	black/green biofilm from waterline	2012	+
Unknown *	Z8x	HAN19/3	Kobben, Hanko	black/green biofilm from waterline	2012	
Unknown *	Z8x	HAN20/1	Kobben, Hanko	sediments 10 cm under water	2012	
Calothrix sp. *	Z8x	HAN20/3	Kobben, Hanko	sediments 10 cm under water	2012	+
Anabaena sp. *	Z8x	HAN21/1	Kobben, Hanko	Gastropod 10 cm under water	2012	+
Unknown *	Z8x	HAN21/2	Kobben, Hanko	Gastropod 10 cm under water	2012	
Calothrix sp. *	Z8x	HAN21/3	Kobben, Hanko	Gastropod 10 cm under water	2012	+
Calothrix sp. *	Z8x	HAN21/4	Kobben, Hanko	Gastropod 10 cm under water	2012	+
Calothrix sp. *	Z8x	HAN21/5	Kobben, Hanko	Gastropod 10 cm under water	2012	+
Nostoc sp. *	Z8x	HAN22	Kobben, Hanko	biofilm growth on rock waterline	2012	+
Calothrix sp. *	Z8x	HAN22/1	Kobben, Hanko	Black biofilm	2012	+
Unknown *	Z8x	HAN22/2	Kobben, Hanko	Black biofilm	2012	
Nostoc sp. *	Z8x	HAN24/1	Kobben, Hanko	epilithic	2012	+
Tolypothrix sp. *	Z8x	HAN25/1	Kobben, Hanko	epilithic	2012	+
Tolypothrix sp. *	Z8x	HAN26/2	Hanko Casino sea shore, Hanko	Black biofilm	2012	+
Nostoc sp. *	Z8x	HAN27	Hanko Casino sea shore, Hanko	small rock pond	2012	+
Unknown *	Z8x	HAN29/3	Hanko Casino sea shore, Hanko	green biofilm on the rock	2012	
Calothrix sp. *	Z8x	HAN30/2	Hanko Casino sea shore, Hanko	Green biofilm and Gastropod	2012	+
Calothrix sp. *	Z8x	HAN30/3	Hanko Casino sea shore, Hanko	Green biofilm and Gastropod	2012	+
Unknown *	Z8x	HAN31/a	Hanko	biofilm	2012	
Unknown *	Z8x	HAN32/2	Hanko Casino sea shore, Hanko	epilithic	2012	
Calothrix sp. *	Z8x	HAN33/1	Hanko Casino sea shore, Hanko	brown biofilm on the rock	2012	+
Calothrix sp. *	Z8x	HAN33/2	Hanko Casino sea shore, Hanko	Brown/yellow biofilm	2012	+
Calothrix sp. *	Z8x	HAN36/2	Hanko Casino sea shore, Hanko	Biofilm	2012	+

Table S1. *Cont.*

Anabaena sp. *	Z8x	HAN37/1	Hanko Casino sea shore, Hanko	green biofilm	2012	+
Calothrix sp. *	Z8x	HAN37/2	Hanko Casino sea shore, Hanko	green biofilm	2012	+
Calothrix sp. *	Z8x	HAN37/3	Hanko Casino sea shore, Hanko	Green biofilm	2012	+
Calothrix sp. *	Z8x	HAN38/2	Hanko Casino sea shore, Hanko	Red biofilm	2012	+
Unknown *	Z8x	HAN40/1	Hanko Casino sea shore, Hanko	epilithic	2012	
Cyanobium sp.	Z8	HIID A3A	Hiidenvesi, Kirkkojärvi	sediment	1999	
Pseudanabaena sp.	Z8	HIID A13A	Hiidenvesi, Kirkkojärvi	sediment	1999	
Leptolyngbya sp.	Z8	HIID A16A	Hiidenvesi, Kirkkojärvi	sediment	1999	
Leptolyngbya sp.	Z8	HIID A19B	Hiidenvesi, Kirkkojärvi	sediment	1999	
Leptolyngbya sp.	Z8	HIID A25A	Hiidenvesi, Kirkkojärvi	sediment	1999	
Trichormus sp. *	Z8x	HIID B6A	Hiidenvesi, Mustionselkä	sediment	1999	
Synechocystis sp.	Z8	HIID B11B	Hiidenvesi, Mustionselkä	sediment	1999	
Leptolyngbya sp.	Z8	HIID B15A	Hiidenvesi, Mustionselkä	sediment	1999	
Pseudanabaena sp.	Z8	HIID B19	Hiidenvesi, Mustionselkä	sediment	1999	
Phormidium sp.	Z8	HIID B22B	Hiidenvesi, Mustionselkä	sediment	1999	
Pseudanabaena sp.	Z8	HIID C15A	Hiidenvesi, Nummelanselkä	sediment	1999	
Nostoc sp. *	Z8x	HIID C18A	Hiidenvesi, Nummelanselkä	sediment	1999	
Leptolyngbya sp.	Z8	HIID D2A	Hiidenvesi, Kiihkelyksenselkä	sediment	1999	
Trichormus sp. *	Z8x	HIID D3	Hiidenvesi, Kiihkelyksenselkä	sediment	1999	
Anabaena sp. *	Z8x	HIID D7A	Hiidenvesi, Kiihkelyksenselkä	sediment	1999	
Nostoc sp. *	Z8x	XHIID A1	Hiidenvesi, Kirkkojärvi	sediment	1999	
Nostoc sp. *	Z8x	XHIID A6	Hiidenvesi, Mustionselkä	sediment	1999	
Anabaena sp. *	Z8x	XHIID B2A	Hiidenvesi, Mustionselkä	sediment	1999	+
Anabaena sp. *	Z8x	XHIID B6	Hiidenvesi, Mustionselkä	sediment	1999	+
Nostoc sp. *	Z8x	XHIID C1	Hiidenvesi, Nummelanselkä	sediment	1999	

Table S1. *Cont.*

Nostoc sp. *	Z8x	XHIID C2	Hiidenvesi, Nummelanselkä	sediment	1999
Nostoc sp. *	Z8x	XHIID C3	Hiidenvesi, Nummelanselkä	sediment	1999
Nostoc sp. *	Z8x	XHIID C4	Hiidenvesi, Nummelanselkä	sediment	1999
Nostoc sp. *	Z8x	XHIID C5A	Hiidenvesi, Nummelanselkä	sediment	1999
Nostoc sp. *	Z8x	XHIID C5B	Hiidenvesi, Nummelanselkä	sediment	1999
Cylindrospermum sp.*	Z8x	XHIID C12	Hiidenvesi, Nummelanselkä	sediment	1999
Aphanothece sp.	Z8x	XHIID D1	Hiidenvesi, Kiihkelyksenselkä	sediment	1999
Nostoc sp. *	Z8x	XHIID D7	Hiidenvesi, Kiihkelyksenselkä	sediment	1999
Nostoc sp. *	Z8x	XHIID D8	Hiidenvesi, Kiihkelyksenselkä	sediment	1999
Nostoc sp. *	Z8x	XHIID D12	Hiidenvesi, Kiihkelyksenselkä	sediment	1999
Nostoc sp. *	Z8x	XHIID D13	Hiidenvesi, Kiihkelyksenselkä	sediment	1999
Nostoc sp. *	Z8x	XHIID D14	Hiidenvesi, Kiihkelyksenselkä	sediment	1999
Nostoc sp. *	Z8x	116.7.5		lichen	
Nostoc sp. *	Z8x	116.6.23		lichen	
Nostoc sp. *	Z8x	N135.9.1		lichen	
Nostoc sp. *	Z8x	N990622Ш Ilona		lichen	
Tolypothrix sp. *	Z8xS	BIR MGR4	The Gulf of Finland		
Nostoc sp. *	Z8x	N122.4.B		lichen	
Nostoc sp. *	Z8x	N124.3.B		lichen	
Nostoc sp. *	Z8x	N122.4B		lichen	
Nostoc sp. *	Z8x	UK4	Itä-Pakila, Helsinki, Finland	lichen *Peltigera spuria*	
Nostoc sp. *	Z8x	UK18AШ	Autti, Finland	lichen *Peltigera leucophlebia*	
Nostoc sp. *	Z8x	N115.3.2		lichen	
Nostoc sp. *	Z8x	UK1	Oulunkylä, Helsinki, Finland	lichen *Peltigera* sp.	

Table S1. *Cont.*

Nostoc sp. *	Z8x	N990653.17		lichen		
Nostoc sp. *	Z8x	102I		lichen		
Nostoc sp. *	Z8x	N123.4		lichen		
Nostoc sp. *	Z8x	N107.3		lichen		
Nostoc sp. *	Z8xS	BIR L1S7	The Gulf of Finland			
Anabaena cylindrica *	Z8x	BIR JV1	The Gulf of Finland			+
Calothrix sp. *	Z8x	UHCC 0022				
Nostoc sp. *	Z8x	N138		lichen		
Nostoc sp. *	Z8x	UK7BIIm	Itä-Pakila, Helsinki, Finland	Lichen *Peltigera canina?*		
Nostoc sp. *	Z8x	UK35		lichen		
Nostoc sp. *	Z8x	UK18AIV	Autti, Finland	Lichen *Peltigera leucophlebia*		
Nostoc sp. *	Z8x	UK18AI	Autti, Finland	Lichen *Peltigera leucophlebia*	2006	+
Nostoc sp. *	Z8x	UK18BI	Autti, Finland	Lichen *Peltigera leucophlebia*		
Nostoc sp. *	Z8x	UK18BIV	Autti, Finland	Lichen *Peltigera leucophlebia*		
Nostoc sp. *	Z8x	UK18BIII	Autti, Finland	Lichen *Peltigera leucophlebia*		
Nostoc sp. *	Z8x	UK2aImI	Oulunkylä, Helsinki, Finland	Lichen *Peltigera praetextata*		+
Nostoc sp. *	Z8x	UK18BII	Autti, Finland	Lichen *Peltigera leucophlebia*		
Nostoc sp. *	Z8x	XPORK5A	Porkkala cape	epiphytic	1999	+
Anabaena sp. *	Z8x	XPORK6B	Porkkala cape	sediment		
Tolypothrix sp. *	Z8x	XPORK34B	Porkkala cape			
Nostoc sp. *	Z8x	Nostoc 152	Lake Sääksjärvi, Finland			AJ133161
Calothrix sp. *	Z8x	Cal 336/2	Vihti, Enäjärvi Laukilanlahti			
Nostoc sp. *	Z8x	N159	Lake Haukkajärvi, Finland			
Tolypothrix sp. *	Z8x	Tol 328	Kuopio, Finland	greenhouse soil	1999	
Calothrix sp. *	Z8x	Cal 336/3	Lake Enäjärvi, Finland	Lake water	1999	

Table S1. *Cont.*

Other strains						
Anabaena cf. Cylindrica *	Z8x	PH133	Lake Arresø, DK		1993	AJ293110
Scytonema sp. *	Z8x	PCC7110	Bermuda	Limestone	1971	
Tolypothrix sp. *	Z8x	PCC7415	Stockholm, Sweden	greenhouse soil	1972	
Calothrix sp. *	Z8x	PCC7103	United States	Herbarium specimen of *Anacystis montana*		
Calothrix sp. *	Z8x	PCC7102	Antofagasta, Chile	sand	1958	
Tolypothrix sp. *	Z8x	PCC7101	Borneo	soil	1950	
Tolypothrix sp. *	Z8x	PCC7504	Stockholm, Sweden	aquarium	1972	
Anabaena cylindrica *	Z8x	PCC7122	Cambridge, UK	water, most likely pond	1939	AF091150
Anabaena sp. *	Z8x	PCC7108	Moss Beach, California USA	interdidal zone	1970	AJ133162
Anabaena variabilis *	Z8x	ATCC29413	Mississippi, USA	Freshwater	1964	NR_074300
Calothrix sp. *	Z8x	PCC7103	unknown	herbarium specimen of Anacystis montana		AM230700
Calothrix sp. *	Z8x	PCC7507	near Kastanienbaum, Vierwaldstättersee, Switzerland	sphagnum bog	1972	NR_102891
Cylindrospermopsis raciborskii *	Z8	CS-505	Queensland, Australia	waterbodies Solomon Dam	1996	EU552055
Cylindrospermum stagnale *	Z8x	PCC7417	Stockholm, Sweden	soil, greenhouse	1972	NR_114701
Nostoc punctiforme *	Z8x	PCC73102	Australia	root section, Macrozamia sp.	1973	AF027655
Nostoc sp. *	Z8x	PCC7120	unknown	unknown	unknown	NR_074310
Fischerella sp. *	Z8x	PCC9339	unknown	unknown	unknown	AB075984
Anabaena sp. *	Z8x	6 sf Calc	Dobre Pole	unknown	1998	

Culture medium containing nitrogen: Z8, BG50, BG11, AA, ASM; Culture medium without nitrogen: Z8x, Z8xS (Z8 without nitrogen plus salt), BG0. * Nostocales or Stigonematales Order which are able to produce heterocyst. + Cyanobacterial strains classified based on 16S rRNA sequences not available in the NCBI database.

Table S2. ^{1}H and ^{13}C NMR data for modified 7-OMe-Scytophycin B (**2**, conformers A and B), unknown 7-OMe-Scytophycin B (C) and 7-OMe-Scytophycin B (**1**, conformers D and E) in [D6] DMSO. ( ) = weak signal, [ ] = split signal.

No	δ_C	δ_H	mult., *J* (Hz)	COSY	HMBC (H→C)
1	165.3	-		-	
2	116.8	5.68	d 15.8	3	1, [2], 3, 4, 5
3	147.4	7.31	d 15.8	2	1, 2, [3], 4, 4-CH$_3$, 5
4	133.9	-		-	
4-CH$_3$	11.7	1.75	s	-	3, 4, [4-CH$_3$], 5, 6
5	136.3	5.80		6, 6′	3, 4, 4-CH$_3$, 6, 7
6	31.9	2.17		5, 6′	(3), 4, 5, 7, 8
6′		2.69		5, 6	(4, 5, 7, 8)
7	77.6	3.45		6, 6′, 8, 8′	7-OCH$_3$, (5, 8, 9)
7-OCH$_3$	55.8	3.32	s	-	7, [7-OCH$_3$]
8	38.3	1.31		7, 8′, 9	6, 7, (9), 10
8′		1.59		(7), 8, 9	6, 7-OCH$_3$, 9, 10
9	69.6	4.30		8, 8′, 10, 11	(8), 10, 11, (13)
10	129.9	5.63		9, 11, 12′	9, [10], 11, 12
11	124.0	5.77		9, 11, 12	9, 10, [11], 12, 13
12	30.3	1.84		11, 13	10, 11, 13
12′		1.87		10, 13	10, 11, 13
13	65.3	3.31		12′, 14′	9, 11, (14), 15
14	34.8	1.42		14′, 15	12, 13, 15, 16
14′		1.50		13, 14, 15	12, 13, 15, 16
15	77.7	3.67	dd 4.4, 6.2	14, 14′	13, 14, 15-OCH$_3$, 16, 16-CH$_2$, 17
15-OCH$_3$	56.8	3.31	s	-	15, [15-OCH$_3$]
16	60.1	-		-	
16-CH$_2$	45.0	2.56		16′-CH$_2$	15, 16, [16-CH$_2$], 17
16′-CH$_2$		2.65		16-CH$_2$	15, 16, [16-CH$_2$], 17
17	75.1	3.53		18, 18′	15, 16, 16-CH$_2$, 17-OCH$_3$, 18, 19
17-OCH$_3$	53.4	3.14	s	-	17, [17-OCH$_3$]
18	28.2	1.37		17, 18′, 19	16, 17, 19, 20
18′		1.73		17, 18, 19	16, 17, 19, 20
19	75.8	3.07		18, 18′	(17), 18, 19-OCH$_3$, 20, 20-CH3, 21
19-OCH$_3$	56.6	3.10	s	-	19, [19-OCH$_3$]
20	37.8	1.87		21, 20-CH$_3$	(18), 20-CH$_3$, 21
20-CH$_3$	9.2	0.79		20	19, 20, [20-CH$_3$], 21
21A	71.9	5.28		20	1, 19, 20, 20-CH$_3$
21B	72.1	5.07	9.5, 19.1	20, 22	1, 19, 20, 22, 22-CH$_3$, 23
22	34.3	2.03		21B, 22-CH$_3$, 23	20, 22-CH$_3$, 23
22-CH$_3$	11.3	0.99		22	21,22, [24-CH$_3$], 23
23	79.2	2.97		22, 24	21, 22, 22-CH$_3$, 24, 24-CH$_3$, 27
24	30.3	1.87		24-CH$_3$	24-CH$_3$, 25
24-CH$_3$	17.3	0.76		24	23, 24, [24-CH$_3$]
25	30.3	1.60			23, 24, 24-CH$_3$, 26, 27ABC
26	27.8	1.27			25, 27

Table S2. *Cont.*

26′		1.49			27, 28, 31AB
27A	84.5	-			
27B	84.3	-			
27C	84.0	-			
27D,E	213.4	-			
28A	48.0	1.62		29A, 28-CH_3A	26, 27A, 28-CH_3A, 29A
28B	48.0	1.68		29B, 28-CH_3B	26, 27B, 28-CH_3B, 29B, (30B)
28CDE	48.0	1.68			
28-CH_3A	7.8	0.76		28A	27A, 28A, [28-CH_3A], 29A
28-CH_3B	7.8	0.77		28B	27B, 28B, [28-CH_3B], 29B
28-CH_3DE	12.9	0.86			[28DE], 29DE
29A	86.0	3.35		28A, 30A	27A,B; (28, 28-CH_3), 29-OCH_3, 30-CH_3, 31A,B
29B	86.1	3.33		28B, 30B	27B, (28, 28-CH_3), 29-OCH_3, 30-CH_3, 31B
29C	84.0	3.31			27C, 30-CH_3C, 31C
29D,E	85.7	3.03			30-CH_3DE, 31D
29-OCH_3	59.7	3.15	s	-	29, [29-CH_3]
30A	35.6	2.46		29A, 30-CH_3A, 31A	31A, 30-CH_3A, 32A
30B	36.2	2.40		29B, 30-CH_3B, 31B	27B, (28B), 29B, 31B, 30-CH_3, 32B
30D	36.3	2.40			(31D)
30E	36.5	2.40			(31E)
30-CH_3 A	15.8	0.97		30A	29AB, 30AB, [30-CH_3 AB], 31AB
30-CH_3 B	15.8	0.96		30B	29AB, 30AB, [30-CH_3 AB], 31AB
30-CH_3 C	15.0	0.90			27C, 29C, [30-CH_3C], 31C
30-CH_3 DE	19.4	1.06			29DE, 30DE, [30-CH_3DE], 31DE
31A	52.9	2.01		30A, 32A	27A, 28, 29A, 30A, 32A
31B	52.9	2.04		30B, 32B	27B, 28, (28-CH_3), 29B, 30B, 30-CH_3, 32B
31C	52.1	2.17		32C	27C, 28, 29C, 30-CH_3 C, 32C, 33-NCH_3 C
31D	110.6	4.97, 5.00		32D, 2.37	30-CH_3 D,E , 32D
31E	112.7	5.07, 5.10		32E, 2.4	-
32A	83.2	5.19	d 2.6	31A	27A, 30A, 31A, 33-NCH_3 A, 34A
32B	89.4	4.50	d 4.0	31B	27B, 30B, 31B, [32B], 33-NCH_3 B, 34B
32C	90.3	4.25	d 9.9	31C	27C, 31C, 33-NCH_3 C, 34C
32D	128.5	6.72, 6.76	d 14.3	31D	30D, 31D, 33-NCH_3 D, 34D
32E	123.7	6.95, 7.01	d	31E	30E, 31E, [32E], 33-NCH_3 E
33-NCH_3 A	28.6	2.86	s	-	32A, [33-NCH_3 A], 34A
33-NCH_3 B	25.7	2.72	s	-	32B, [33-NCH_3 B], 34B
33-NCH_3 C	24.3	2.61	s	-	32C, [33-NCH_3 C], 34C
33-NCH_3 D	26.5	2.86	s	-	32D, [33-NCH_3 D], 34D
33-NCH_3 E	32.3	2.98	s	-	32E, [33-NCH_3 E], 34E
34A	163.7	8.18	s	-	32A, 33-NCH_3 A, [34A]
34B	162.2	8.26	s	-	32B, 33-NCH_3 B, [34B]
34C	163.2	8.22	s	-	32C, 33-NCH_3 C, [34C]
34D	162.3	8.34	s	-	32D, 33-NCH_3 D, [34D]
34E	161.1	8.07	s	-	32E, 33-NCH_3 E, [34E]

Table S3. Scytophycin variants (No and Name), retention times (R_t), experimental (Exp) and calculated (Cal) ion masses (*m*/*z*) of $[M + H − H_2O]^+$, $[M + Na]^+$ and $[hk − 4H + Na]^+$, mass error (**Δ**) in ppm, molecular formula and frequency in strains HAN3/2 = *Scytonema* sp. HAN3/2, HAN11/1 = *Nostoc* sp. HAN11/1, HAN21/1 = *Anabaena* sp. HAN21/1 and PH133 = *Anabaena cf. cylindrica* PH133. hk = product ion of the sodiated scytophycin, see Figure S8. Accurate mass measurements obtained with Q-TOF and others with ion trap.

			(*m*/*z*)					(*m*/*z*)				Strain			
	**Variant**	**R_t**	**$[M + H − H_2O]^+$**	**$[M + Na]^+$**		**Δ**		**$[hk − 4H + Na]^+$**		**Δ**		**HAN3/2**	**HAN11/1**	**HAN21/1**	**PH133**
**No**	**Name (Sc = scytophycin)**	**(min)**		**Exp**	**Cal**	**(ppm)**	**Formula**	**Exp**	**Cal**	**(ppm)**	**Formula**				
1	6-OH-7-OMe-Sc	18.1	848.5	888.5					620.4133			−	+	−	−
2	6-OH-7-OMe-17-OH-Sc-D/E	18.9	820.5	860.5					608.4133			−	+	−	−
3	6-OH-7-OMe-Sc	18.9	864.5	904.5					652.4031			−	+	−	−
4	Sc	19.3	774.5	814.5								+	−	−	−
5	6-OH-Sc-D/E	19.3	820.5	860.5					622.4289			−	+	−	−
6	6-OH-Sc-B	19.4	818.5	858.4951	858.4974	-2.69	$C_{45}H_{73}NO_{13}$		620.4133			+	−	−	+
7	Sc	19.4	834.5	874.5								+	−	−	−
8	6-OH-7-OMe-Sc-D/E	19.5	850.5	890.5					638.4239			−	+	−	−
9	Sc	19.8	818.5	858.5					636.4082			−	−	−	+
10	6-OH-7-OMe-15-O-deMe-Sc-B	19.9	818.5	858.5					606.4340			−	++	−	−
11	Sc	20.0	774.5	814.5								+	−	−	+
12	Sc	20.0	804.5	844.5					606.3976			+	+	−	−
13		20.0	834.5	874.5								+	−	−	−
14	29-OH-Sc-B	20.1	788.5	828.5					606.3976			+	−	−	−
15	6-OH-7-OMe-Sc-D/E	20.1	834.5	874.5					622.4289			−	++	−	−
16	X-OH-Sc-D/E (not 6-OH)	20.3	820.5	860.5116	860.5131	−1.70	$C_{45}H_{75}NO_{13}$		638.4239			++	−	+	++
17	Sc	20.4	834.5	874.5					652.4395			+	−	−	+
18	Sc-D/E	20.6	804.5	844.5157	844.5181	−2.90	$C_{45}H_{75}NO_{12}$	622.3926	622.3926	0.08	$C_{32}H_{57}NO_9$	++	−	+	++
19	Sc	20.6	814.5	-								−	++	−	−
20	6-OH-7-OMe-Sc-B	20.6	832.5	872.5					620.4133			−	+++	+	−
21	Sc	20.8	788.5	828.4861	828.4868	−0.90	$C_{44}H_{71}NO_{12}$	606.3602	606.3613	−1.74	$C_{31}H_{53}NO_9$	+	−	+	+
22	Sc	20.9	802.5	842.5								++	−	+	−

Table S3. *Cont.*

23	Sc	21.0	804.5	844.5					622.4289			+	+	+	+
24	Sc	21.2	774.5	814.5063	814.5076	−1.58	$C_{44}H_{73}NO_{11}$	592.3782	592.3820	−6.40	$C_{31}H_{55}NO_8$	+	+	+	−
25	Sc-B	21.3	802.5	842.5022	842.5025	−0.35	$C_{45}H_{73}NO_{12}$	620.3766	620.3769	−0.49	$C_{32}H_{55}NO_9$	+++	++	++	+++
26	Sc	21.9	786.5	826.5062	826.5076	−1.67	$C_{45}H_{73}NO_{11}$	604.3822	604.3820	0.35	$C_{32}H_{55}NO_8$	++	+	+	++
27	Sc-C	22.4	788.5	828.5221	828.5232	−1.37	$C_{45}H_{75}NO_{11}$	606.3971	606.3976	−0.89	$C_{32}H_{57}NO_8$	++	++	+	++
28	7-OMe-Sc	22.5	834.5	874.5								−	−	+	−
29	7-OMe-29-OAc-Sc-B	22.6	844.5	884.5129	884.5131	−0.18	$C_{47}H_{75}NO_{13}$	648.3705	648.3718	−2.03	$C_{33}H_{55}NO_{10}$	−	−	++	−
30	7-OMe-Sc-B (1)	23.5	816.5	856.5	856.5181				620.4133			+	+	+++	+++
31	7-OMe-Sc	24.0	800.5	840.5	840.4868				604.3820			−	+	++	+
32	Sc	24.8	743.5	783.5	783.4654				561.3762			+	−	−	+
33	7-OMe-Sc-C	24.9	802.5	842.5	842.5389				606.4340			−	+	+	+

Table S4. Previously published scytophycin variants, their monoisotopic masses (M), positions of the variable functional groups. 6-OH-7-OMe-scytophycin B = tolytoxin A. Carbon numbers in Figure S8.

No	Name	M	C-6	C-7	C-16	C-19	C-23	C-27	Reference
1	Scytophycin A	821.5	-H	-OH	-epoxy	-OMe	-OH	-OH	[1]
2	Scytophycin B	819.5	-H	-OH	-epoxy	-OMe	-OH	=O	[1]
3	Scytophycin C	805.5	-H	-OH	-Me	-OMe	-OH	=O	[2]
4	Scytophycin D	821.5	-H	-OH	-(OH)CH_3	-OMe	-OH	=O	[2]
5	Scytophycin E	821.5	-H	-OH	-CH_2OH	-OMe	-OH	=O	[2]
6	6-OH-7-OMe-scytophycin B	849.5	-OH	-OMe	-epoxy	-OMe	-OH	=O	[3]
7	23-Ac-tolytoxin A	891.5	-OH	-OMe	-epoxy	-OMe	-OAc	=O	[4]
8	6-OH-scytophycin B	835.5	-OH	-OH	-epoxy	-OMe	-OH	=O	[3]
9	7-Ac-scytophycin B	861.5	-H	-OAc	-epoxy	-OMe	-OH	=O	[1]
10	7-OMe-scytophycin B	833.5	-H	-OMe	-epoxy	-OMe	-OH	=O	[5]

Table S4. *Cont.*

11	19-O-deMe-scytophycin C	791.5	-H	-OH	-Me	-OH	-OH	=O	[3]
12	6-OH-7-OMe-scytophycin E	851.5	-OH	-OMe	-CH_2OH	-OMe	-OH	=O	[3]
13	6-OH-scytophycin D/E	837.5	-OH	-OH	-(OH)CH_3/-CH_2OH	-OMe	-OH	=O	[5]
14	7-OMe-19-deMe-scytophycin D/E	821.5	-H	-OMe	-(OH)CH_3/-CH_2OH	-OH	-OH	=O	[5]

Table S5. Sodiated ion $[M + Na]^+$ mass (*m/z*), product ions and their *m/z*, intensities and comments concerning the tentative structures of scytophycin variants No 1–33. Ion structures a–n are presented in Figure S8.

		Diagnostic Product Ions (x if Same As in the Column Header) and Intensities (Superskripts) from $[M + Na]^+$ (*m/z*)																
**No**	**$[M+Na]^+$ (*m/z*)**	**a-2H 262**	**b-2H 320**	**c-H_2O 332**	**dk-2H 390**	**ek-4H 460**	**fk-4H 490**	**gk-(2H + H_2O) 560**	**hk−4H 620**	**ik−4H 686**	**jk−2H 718**	**l 545**	**lhk−4H 323**	**m 687**	**mhk−4H 465**	**n 731**	**In addition**	**Comment (+ and – Compared to Sc No25)**
1	888.5	x^{3}	x^{26}	x^{1}	x^{9}	x^{6}	x^{7}	x^{6}	x^{100}	660^{30}	732^{3}	591^{13}	x^{2}	733^{3}	x^{1}	777^{1}	-	
2	860.5	x^{1}	x^{19}	x^{1}	x^{6}	446^{1}	476^{14}	548^{2}	608^{100}	720^{8}	734^{1}	563^{5}	x^{-}	705^{1}	453^{1}	749$^{<1}$	-	
3	904.5	x^{1}	x^{18}	x^{3}	x^{7}	x^{5}	x^{14}	x^{-}	652^{100}	718^{1}	764^{1}	x^{-}	x^{2}	749^{2}	497^{1}	793^{1}	-	+2 x O in C13–34
4	814.5	x^{2}	x^{25}	x^{2}	x^{15}	x^{-}	-	532^{1}	592^{100}	658^{1}	736^{3}	517^{2}	295^{1}	673^{100}	-	703^{-}	-	−28 Da
5	860.5	x^{2}	x^{15}	x^{1}	x^{5}	x^{2}	x^{3}	562^{1}	622^{100}	688^{5}	x^{1}	563^{7}	325^{3}	705^{2}	467^{1}	749$^{<1}$	-	
6	858.5	x^{7}	x^{42}	x^{1}	x^{8}	x^{6}	x^{11}	x^{4}	x^{100}	686^{4}	x^{1}	561^{13}	x^{4}	703^{7}	x^{1}	747$^{<1}$	-	
7	874.5	x^{1}	x^{5}	x^{-}	x^{2}	x^{1}	x^{1}	592^{2}	652^{100}	718^{2}	x^{2}	577^{4}	355^{3}	719^{1}	497$^{<1}$	763$^{<1}$	-	+32 Da (2O) in ion lhk
8	890.5	x$^{<1}$	x^{9}	x^{-}	x^{4}	x^{1}	x^{2}	578^{2}	638^{100}	704^{-}	750^{1}	593^{3}	341^{2}	735^{3}	483^{1}	779^{-}	ek-4H: 476^{2}	+16 Da (O) in ion lhk
9	858.5	x^{2}	x^{12}	x^{2}	x^{4}	x^{7}	x^{9}	576^{2}	636^{100}	702^{3}	734^{-}	561^{5}	339^{1}	703^{-}	481^{1}	747^{-}	-	+16 Da (O)
10	858.5	x^{2}	x^{12}	x^{4}	x^{6}	x^{2}	x^{3}	546^{4}	606^{100}	672^{-}	718$^{<1}$	561^{5}	309^{2}	703^{3}	451^{-}	747^{1}	-	16-CH_3
11	814.5	x^{9}	x^{21}	x^{2}	x^{9}	432^{2}	462^{2}	532^{1}	592^{100}	658^{2}	690^{-}	517^{3}	295^{2}	659^{5}	437^{1}	703^{-}	-	−28 Da in ion lhk

Table S5. *Cont.*

12	844.5	x^{9}	x^{51}	x^{2}	x^{16}	444^{1}	476^{3}	546^{3}	606^{100}	688^{7}	704^{OP}	547^{11}	309^{1}	689^{5}	451^{2}	$733^{<1}$	416^{5}, 587^{10}, 703^{20}	Detailed structure not identifiable
13	874.5	x^{3}	x^{15}	$x^{<1}$	x^{3}	x^{7}	x^{5}	x^{1}	x^{100}	x^{2}	x^{5}	577^{3}	x^{1}	719^{-}	$x^{<1}$	763^{-}		+32 in C1-12
14	828.5	$x^{<1}$	x^{13}	x^{1}	x^{3}	$x^{<1}$	x^{3}	546^{5}	606^{59}	671^{2}	704^{1}	x^{op}	x^{1}	687^{100}	x^{29}	717^{-}	306^{2}, 546^{5}, 564^{25}	Spectrum contaminated from ion *m/z* 830.5
15	874.5	x^{3}	x^{11}	x^{3}	x^{5}	x^{1}	x^{3}	562^{2}	622^{100}	$688^{<1}$	734^{2}	577^{5}	325^{2}	719^{1}	467^{1}	763^{OP}		
16	860.5	x^{1}	x^{9}	-	x^{4}	$x^{<1}$	x^{2}	578^{1}	638^{100}	704^{2}	736^{-}	563^{4}	341^{2}	705^{1}	483^{1}	749^{1}		+18 Da (2H, O) in ion lhk
17	874.5	x^{1}	x^{5}	-	x^{2}	x^{1}	x^{1}	592^{2}	652^{100}	718^{2}	750^{-}	577^{4}	355^{3}	719^{1}	$497^{<1}$	763^{-}		+32 Da (2O) in ion lhk
18	844.5	x^{1}	x^{14}	$x^{<1}$	x^{4}	x^{1}	x^{3}	562^{2}	622^{100}	688^{2}	720^{-}	547^{3}	325^{2}	689^{2}	467^{3}	733^{-}		
20	872.5	x^{2}	x^{24}	x^{2}	x^{7}	x^{4}	x^{3}	x^{1}	x^{100}	x^{op}	$732^{<1}$	575^{6}	x^{1}	717^{3}	$x^{<1}$	761^{-}	jk-4H:730^{3}	
21	828.5	x^{3}	x^{25}	$x^{<1}$	x^{9}	x^{3}	x^{5}	546^{3}	606^{100}	672^{2}	704^{-}	531^{5}	309^{2}	673^{3}	451^{1}	717^{-}	562^{65}	-CH_2 from ion lhk
22	842.5	x^{1}	x^{1}	x^{7}	-	x^{1}	x^{4}	x^{2}	x^{100}	x^{1}	x^{-}	x^{-}	x^{-}	x^{OP}	x^{1}	x^{-}		Detailed structure not identifiable
23	844.5	x^{2}	x^{19}	x^{1}	x^{5}	$x^{<1}$	x^{2}	562^{3}	622^{100}	$688^{<1}$	720^{-}	547^{4}	325^{2}	689^{1}	$467^{<1}$	733^{-}		Detailed structure not identifiable
24	814.5	x^{2}	x^{25}	x^{2}	x^{15}	x^{1}	x^{-}	532^{1}	592^{100}	658^{1}	690^{-}	517^{2}	$295^{<1}$	659^{2}	$437^{<1}$	703^{1}		−28 Da (C and O)
25	842.5	x^{3}	x^{15}	$x^{<1}$	x^{4}	x^{4}	x^{4}	x^{3}	x^{100}	x^{1}	x^{1}	x^{3}	x^{2}	x^{1}	$x^{<1}$	$x^{<1}$		
26	826.5	x^{3}	x^{30}	x^{2}	x^{6}	-	-	544^{4}	604^{100}	670^{1}	x^{1}	529^{3}	307^{1}	671^{1}	$449^{<1}$	$715^{<1}$		−16 (O) from C15/16/17/19
27	828.5	x^{3}	x^{22}	x^{1}	x^{6}	x^{2}	x^{1}	546^{1}	606^{100}	672^{2}	704^{-}	531^{2}	309^{1}	673^{OP}	451^{2}	717^{-}		

Table S5. *Cont.*

28	874.5	$x^{<1}$	x^{7}	$x^{<1}$	x^{3}	x^{2}	x^{4}	578^{3}	638^{100}	704^{op}	750^{-}	577^{2}	341^{4}	719^{1}	483^{1}	763^{-}		+18 Da (2H, O) in ion lhk
29	884.5	290^{-}	348^{7}	$360^{<1}$	418^{3}	488^{-}	458^{2}	588^{op}	648^{55}	714^{1}	732^{-}	559^{7}	x^{7}	701^{5}	x^{5}	759^{-}	288^{6}, 528^{17}, 588^{100}	-OAc cleave as HAc (60 Da)
30	856.5	x^{2}	x^{20}	x^{2}	x^{4}	x^{5}	x^{5}	x^{3}	x^{100}	686^{-}	732^{-}	559^{5}	x^{5}	701^{1}	x^{2}	745^{-}		
31	840.5	x^{5}	x^{27}	$x^{<1}$	x^{4}	$x^{<1}$	$x^{<1}$	544^{4}	604^{100}	670^{-}	$716^{<1}$	543^{5}	207^{1}	685^{1}	449^{1}	$x^{<1}$		−16 Da (O) from C15/16/17
32	783.5	203^{-}	261^{-}	$273^{<1}$	331^{2}	401^{6}	431^{7}	501^{1}	561^{100}	627^{2}	659^{1}	x^{-}	x^{1}	$x^{<1}$	$x^{<1}$	x^{-}		−59 Da ($H_3CNHCHO$)
33	842.5	x^{1}	x^{37}	x^{3}	x^{5}	-	x^{2}	546^{op}	606^{100}	$672^{<1}$	x^{1}	x^{2}	309^{1}	687^{1}	$451^{<1}$	x^{-}		

^{OP} = Overlapping peaks.

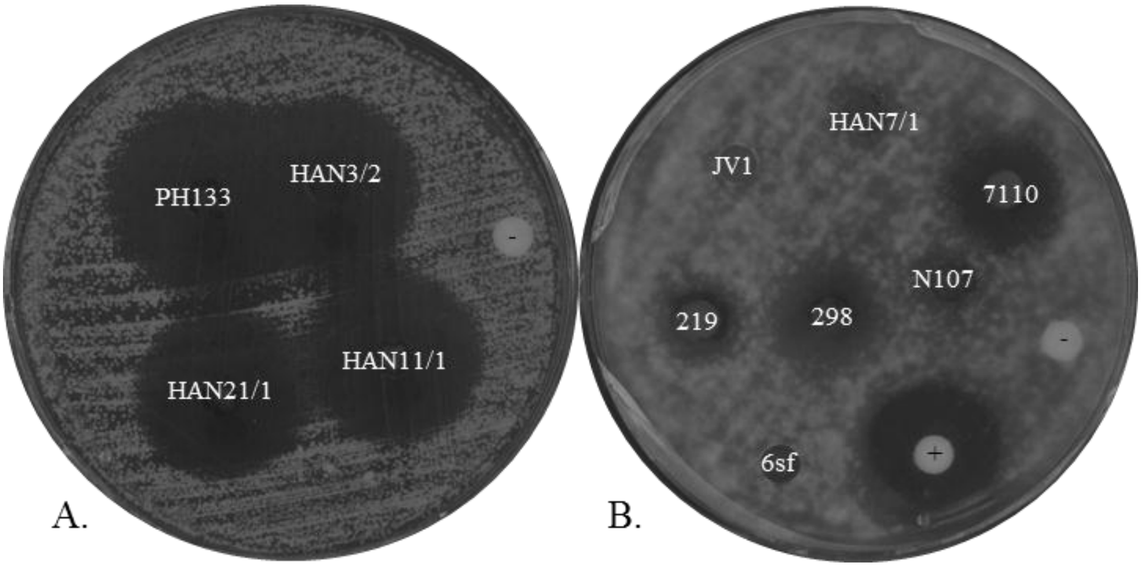

Figure S1. Disc diffusion bioassay using cyanobacterial extracts against *Candida albicans* (**A**) and *Aspergillus flavus* (**B**). PH133 = *Anabaena cf. cylindrica* PH133; HAN3/2 = *Scytonema* sp. HAN3/2; HAN21/1 = *Anabaena* sp. HAN21/1; HAN11/1 = *Nostoc* sp. HAN11/1; HAN7/1 = *Anabaena* sp. HAN7/1; JV 1 = *Anabaena* sp. BIR JV1; 219 = *Nostoc* sp. CENA 219; 6sf = *Nostoc calcicula* 6 sf Calc;298= *Fischerella* sp. CENA 298; N107 = *Nostoc* sp. N107.3; 7110 = *Scytonema hofmanni* PCC 7110; − is negative control with methanol and + is 0.1 mg of nystatin (Sigma-Aldrich, St. Louis, MO, USA).

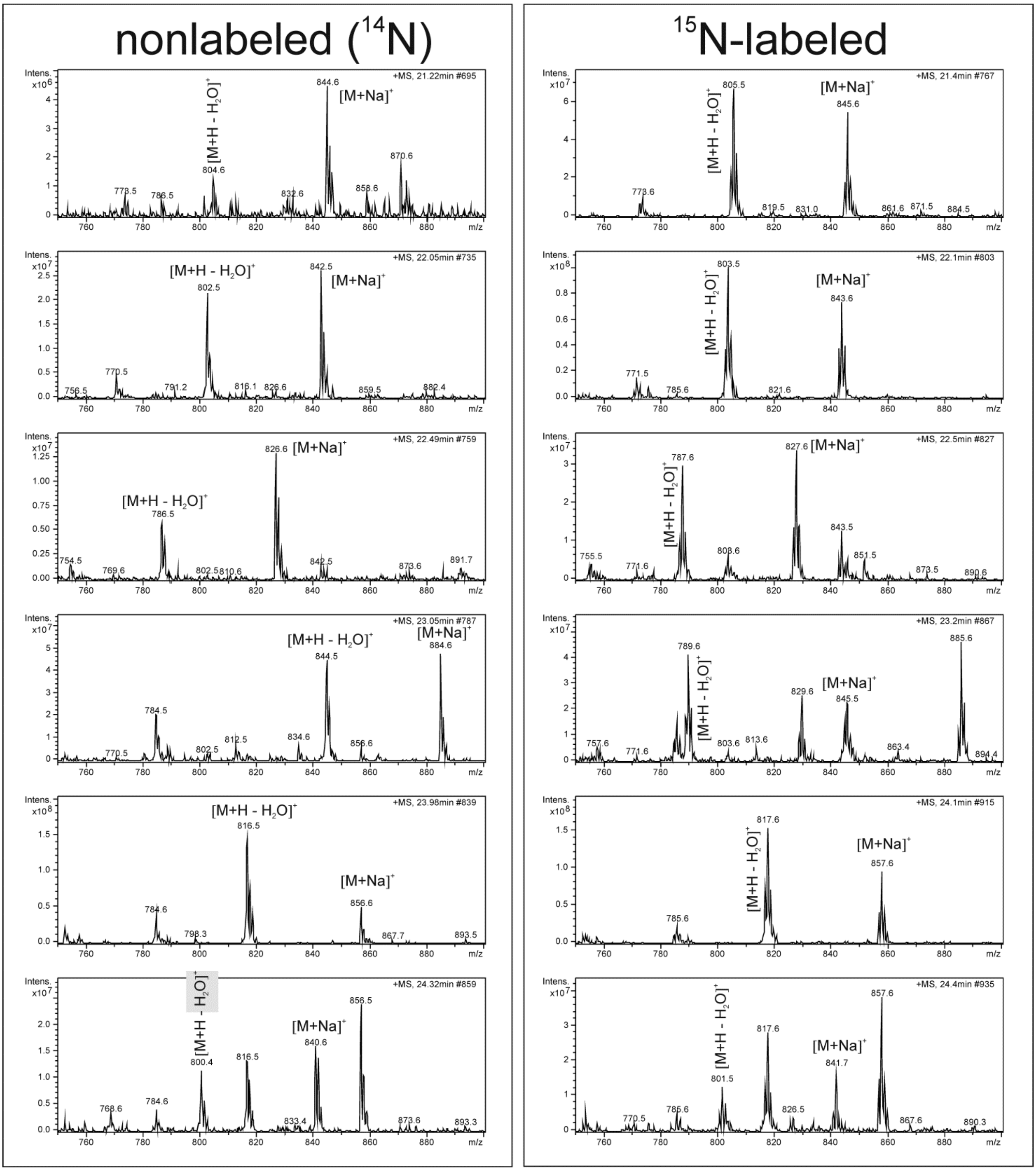

Figure S2. Nonlabeled (^{14}N) and ^{15}N-labeled sodiated and dehydrated protonated scytophycin ions from *Anabaena* sp. HAN21/1.

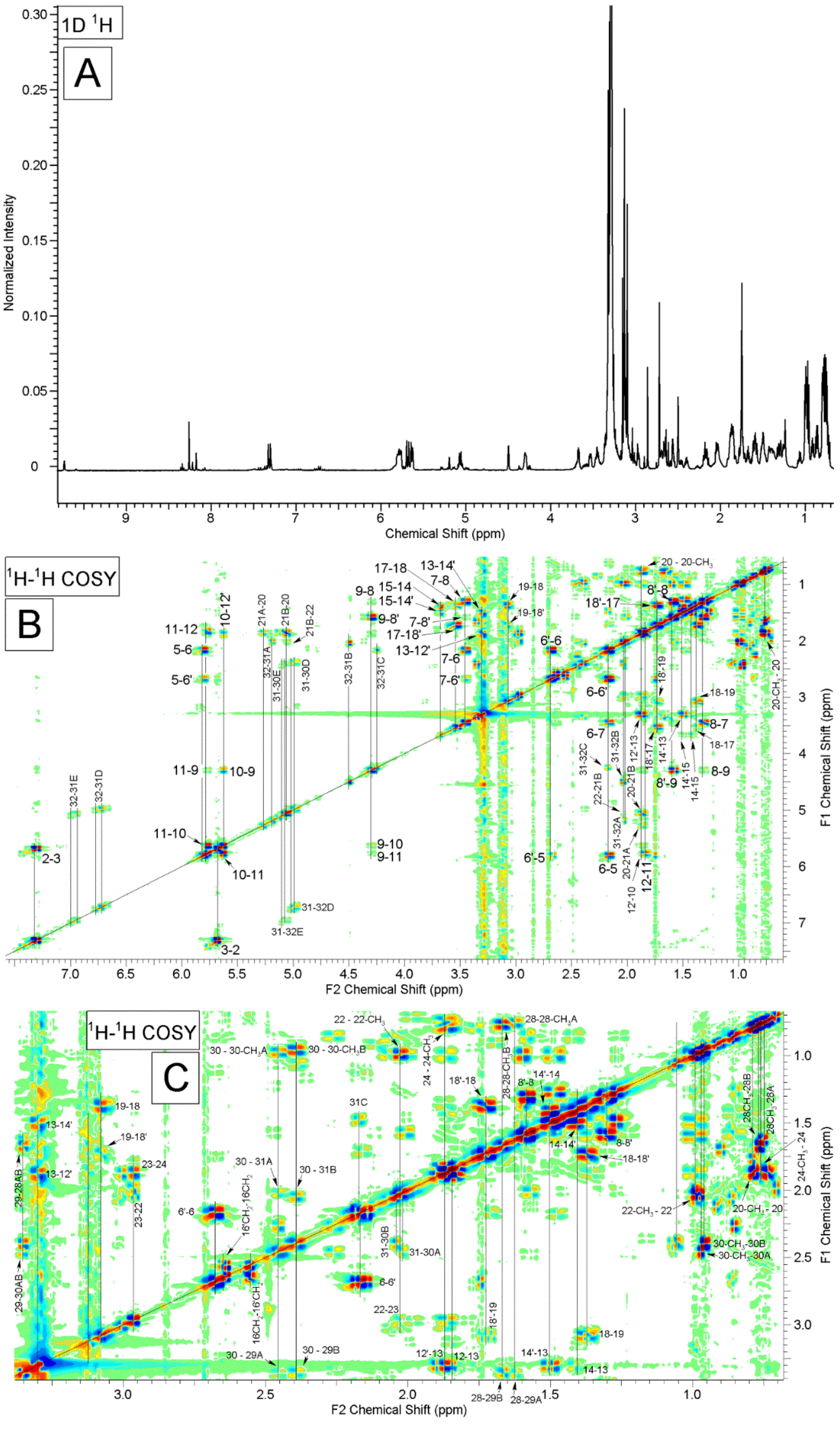

Figure S3. *Cont.*

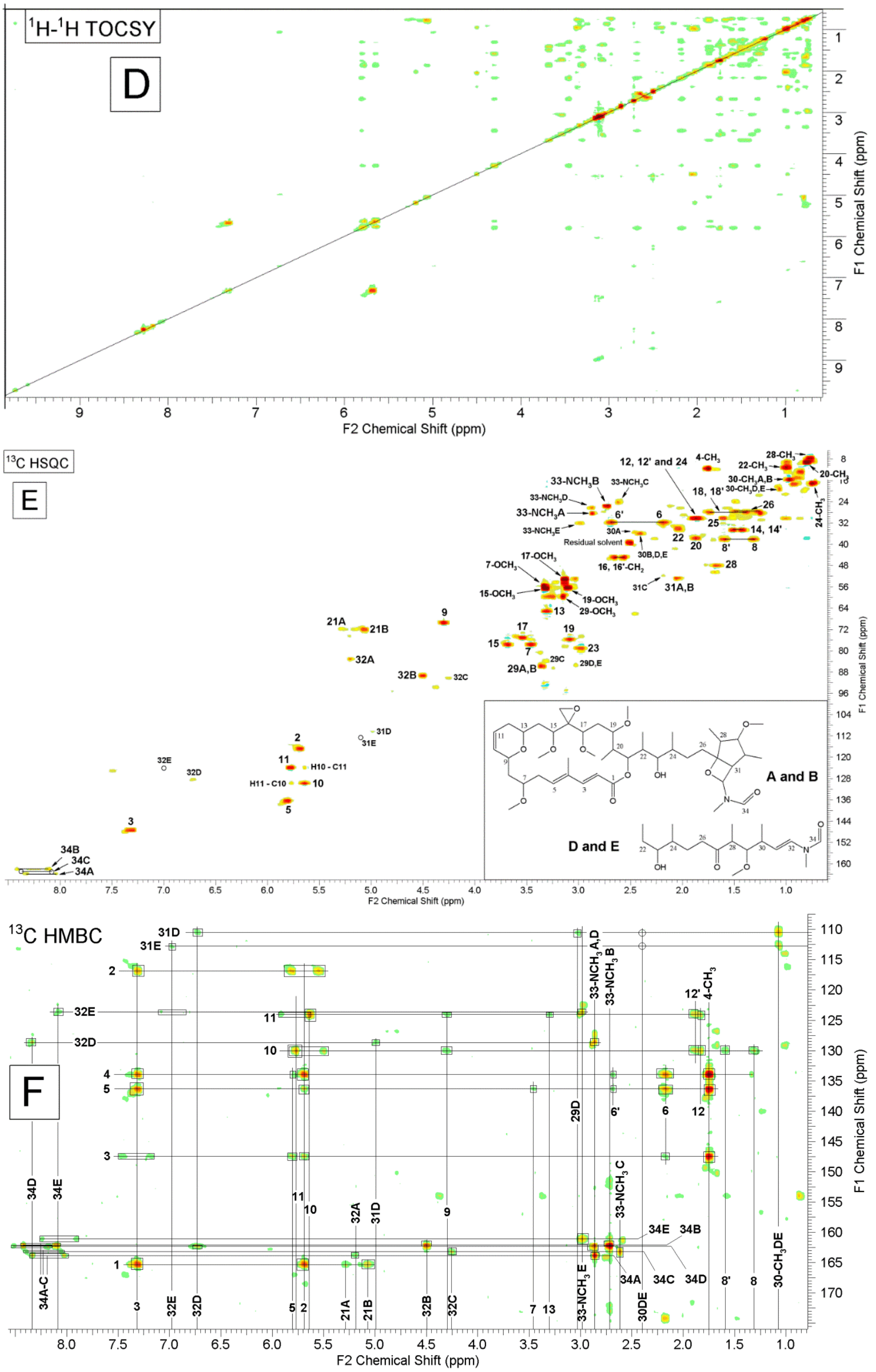

Figure S3. *Cont.*

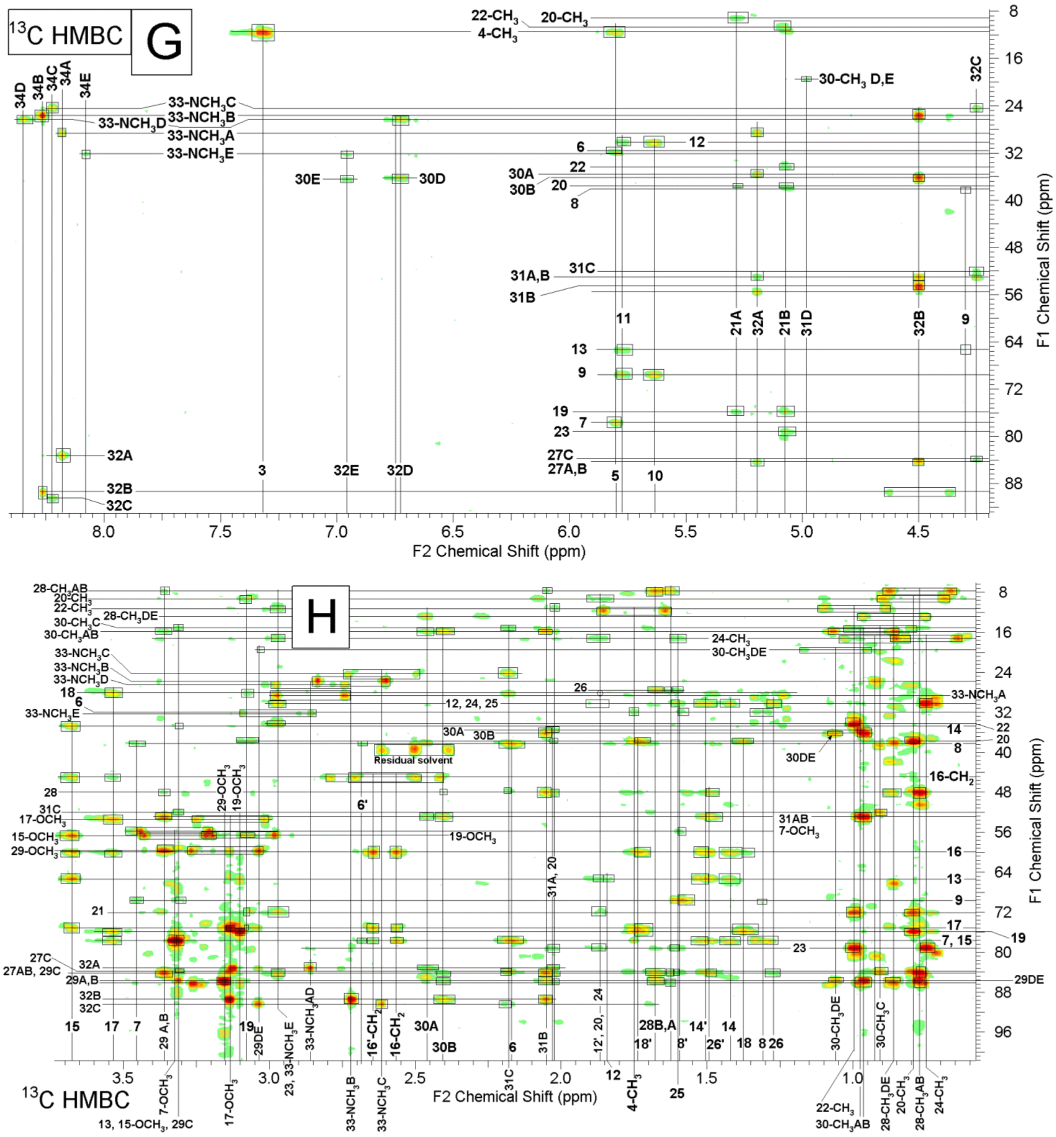

Figure S3. NMR spectra of scytophycin preparate containing mainly 7-OMe-scytophycin-B derivative (**2**, Figure 1) and as a minor component native 7-OMe-scytophycin-B (**1**, Figure 1). ^{1}H NMR spectrum (**A**). Annotated ^{1}H-^{1}H COSY spectra (**B**,**C**). ^{1}H-^{1}H TOCSY spectrum (**D**). Annotated ^{13}C-HSQC spectrum (**E**). Annotated ^{13}C HMBC spectrum (**F**–**H**). ○ = signal intensity too low to be seen in ^{13}C-HSQC spectrum.

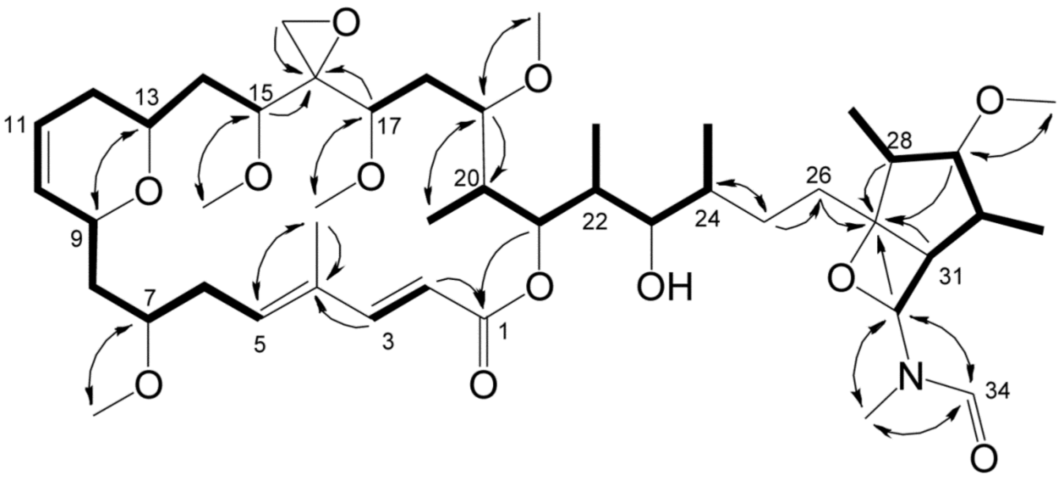

Figure S4. Structure of photoreacted 7-OMe-scytophycin B (**2**, Figure 1) with ^{1}H-^{1}H DQF-COSY (bold lines) and ^{13}C HMBC (arrows) correlations.

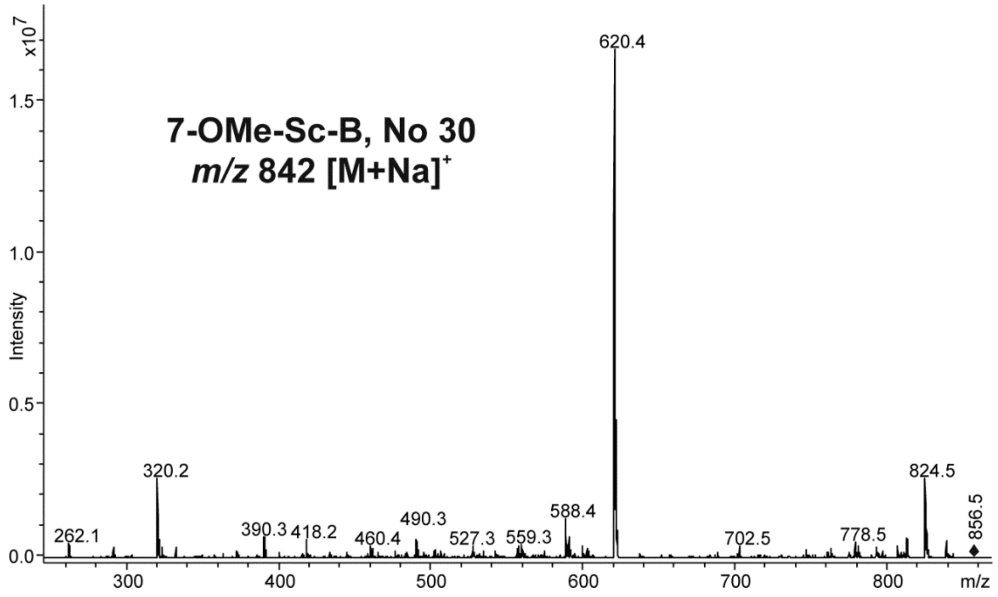

Figure S5. Product ion spectrum from sodiated 7-OMe-scytophycin-B (*m/z* 856.5) produced by *Anabaena* sp. HAN21/1.

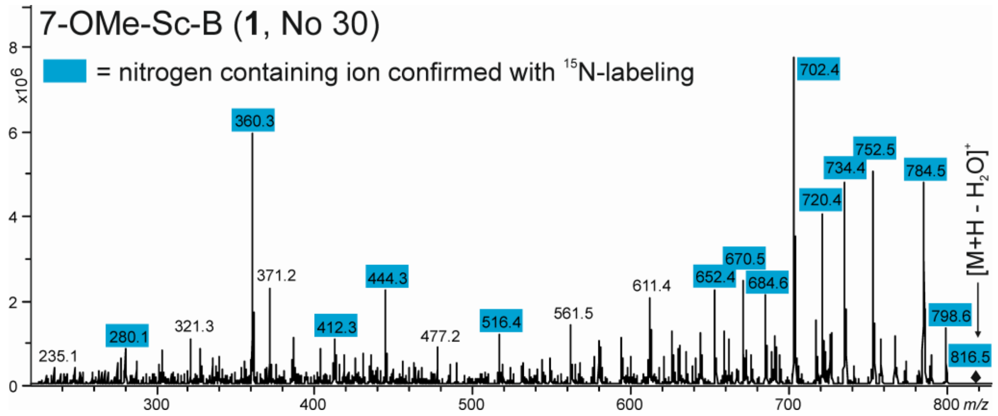

Figure S6. Product ion spectrum from dehydrated protonated 7-OMe-scytophycin-B from *Anabaena* sp. HAN21/1.

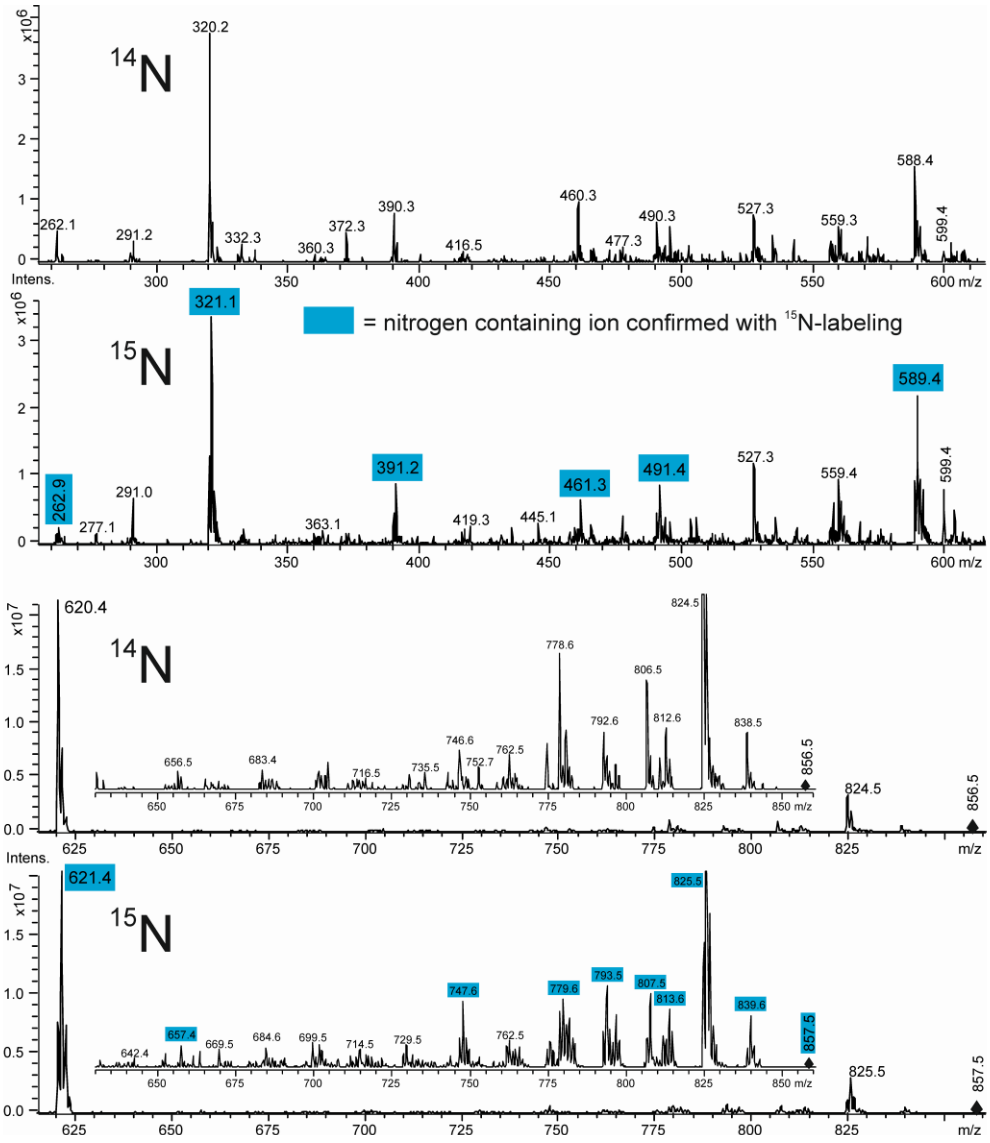

Figure S7. Nonlabeled (^{14}N) and ^{15}N-labeled product ion spectrum from sodiated 7-OMe-scytophycin-B (**1**, Figure 1 and No 30, Table 2) of *Anabaena* sp. HAN21/1.

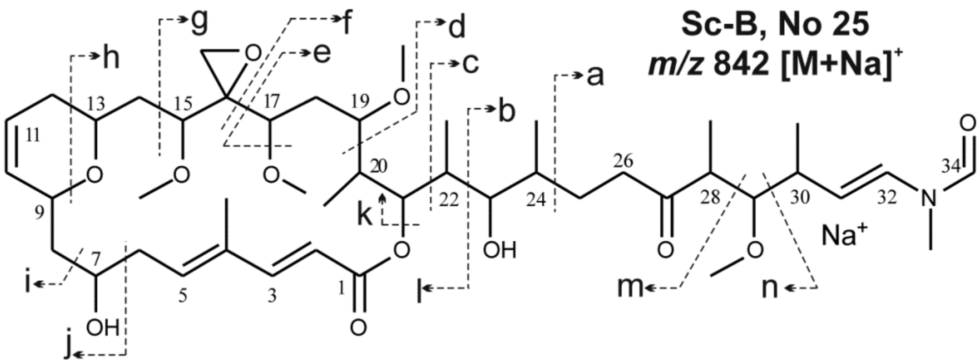

Figure S8. Structure of scytophycin-B (No 25 in Table 2 and Tables S2 and S4) showing the product ions (a, b, c, dk, ek, fk, gk, hk, ik, jk, lhk, l, mhk, m and n) studied from the sodiated ion [M + Na]$^+$.

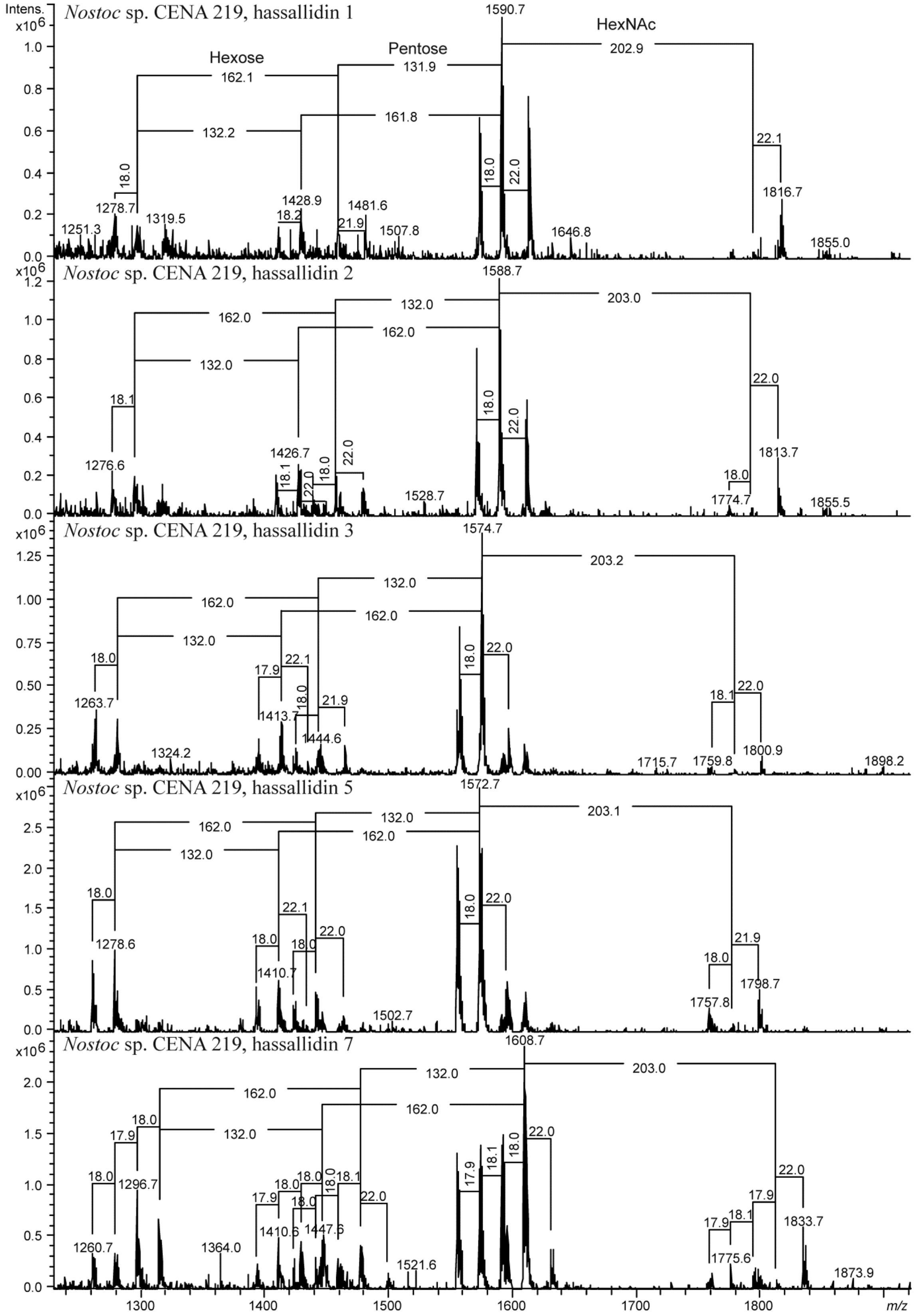

Figure S9. Mass spectra of *Nostoc* sp. CENA 219 hassallidins 1, 2, 3, 5 and 7.

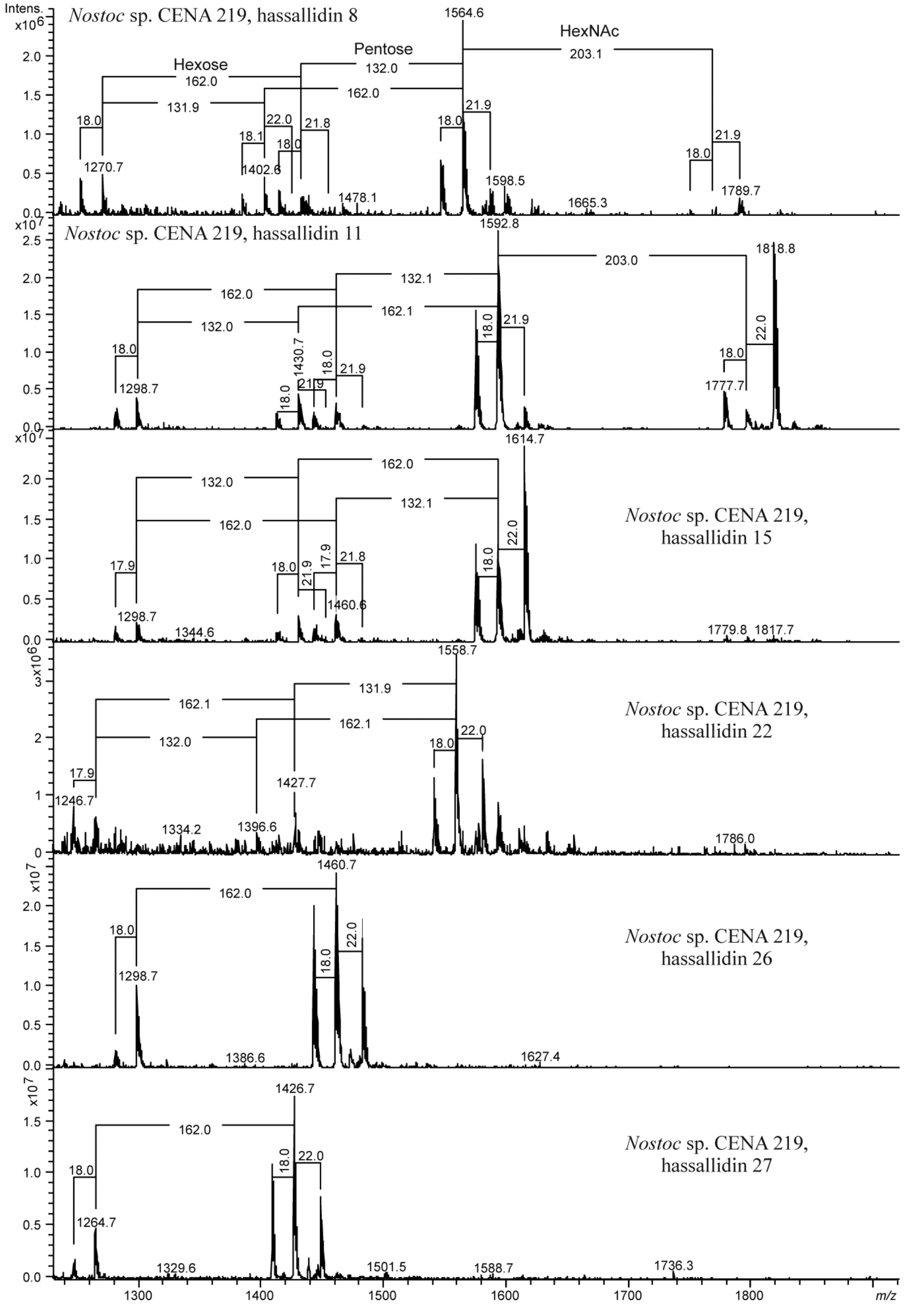

Figure S10. Mass spectra of *Nostoc* sp. CENA 219 hassallidins 8, 11, 15, 22, 26 and 27.

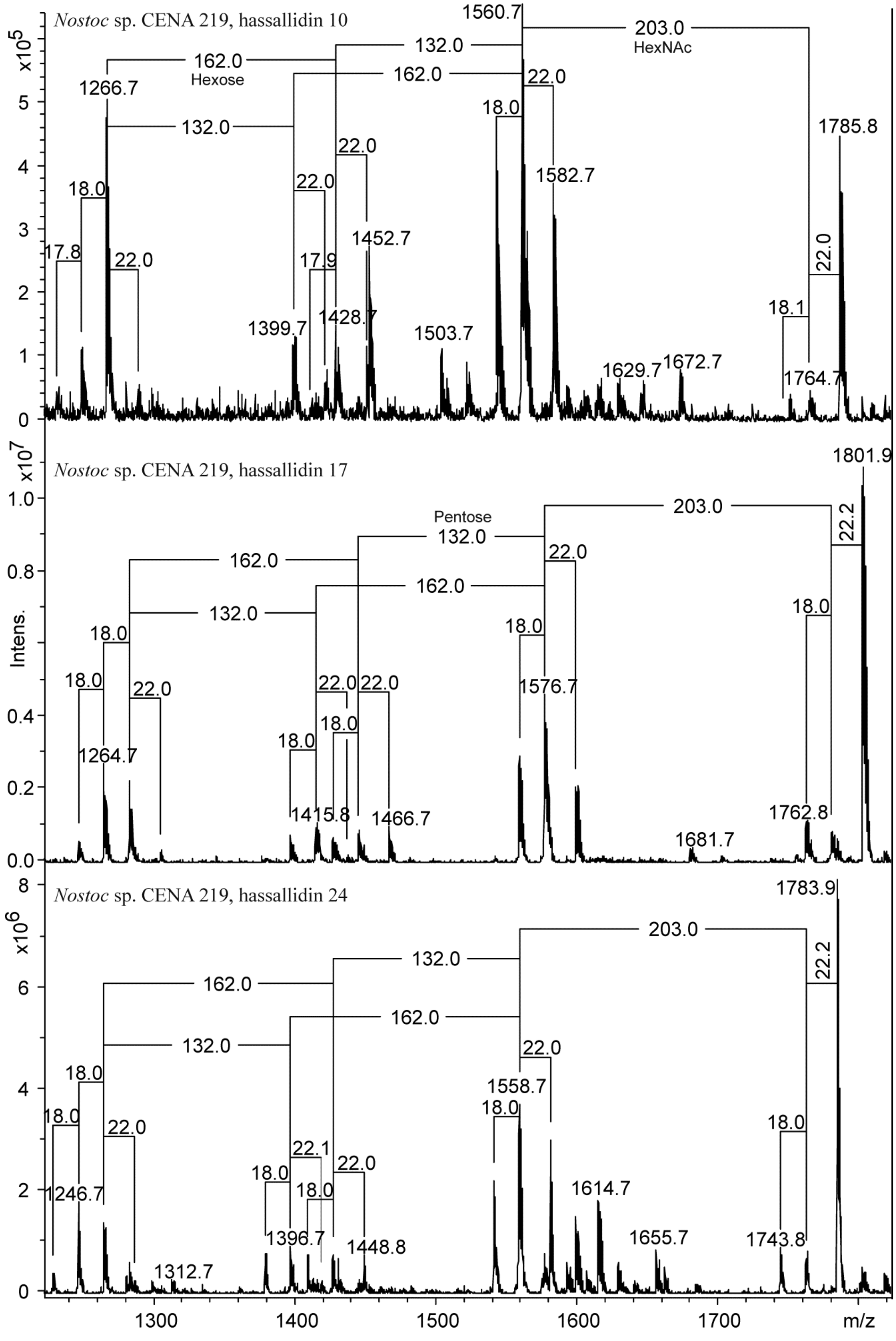

Figure S11. Mass spectra of *Nostoc* sp. CENA 219 hassallidins 10, 17 and 24.

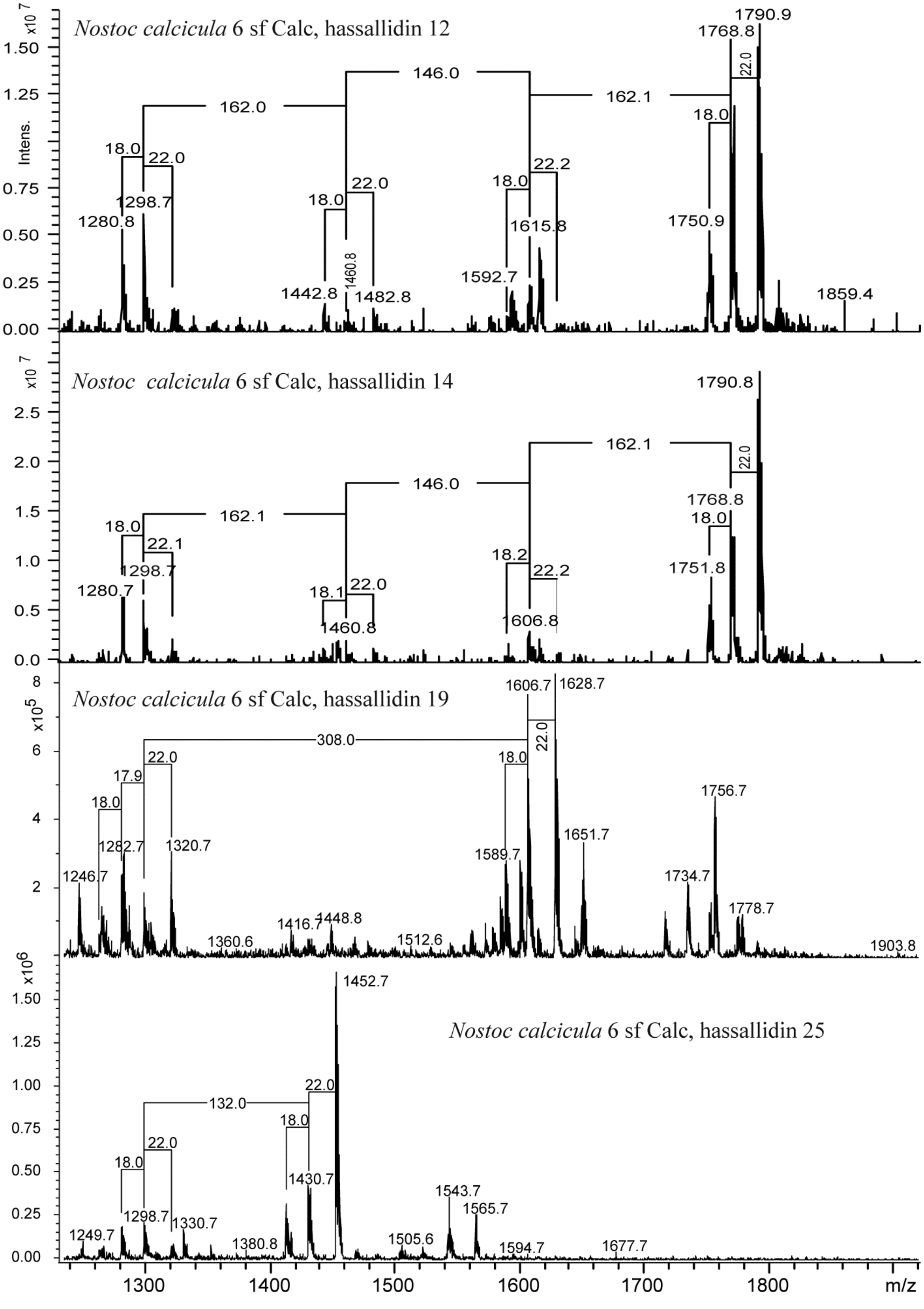

Figure S12. Mass spectra of *Nostoc* sp. 6 sf Calc hassallidins 12, 14, 19 and 25.

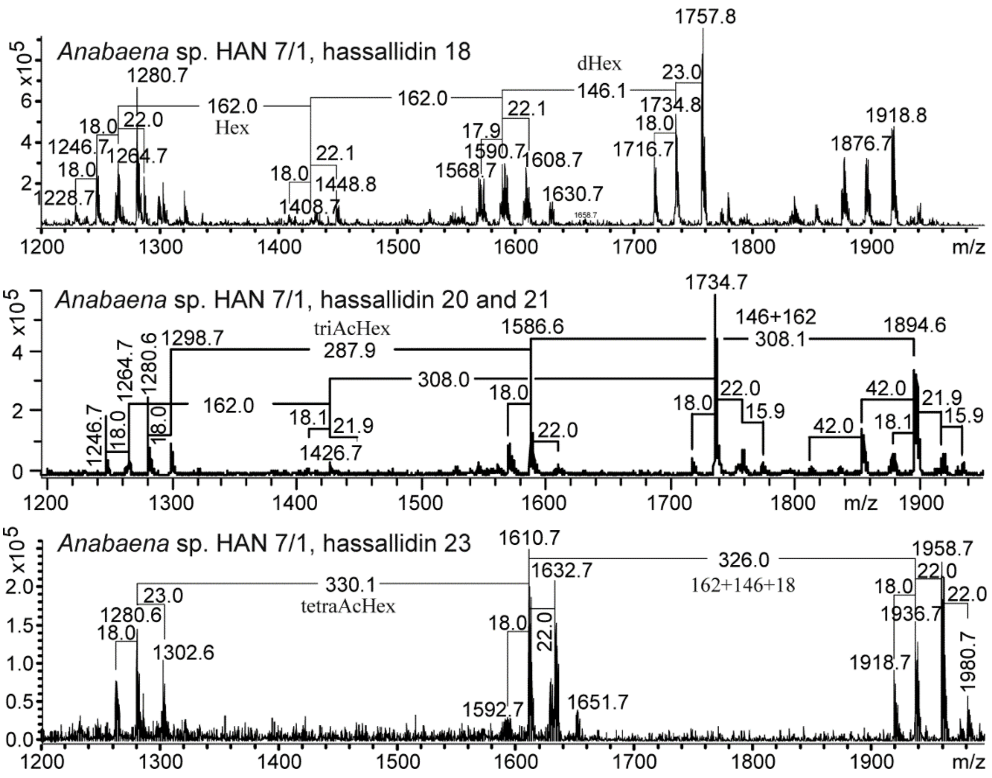

Figure S13. Mass spectra of *Anabaena* sp. HAN7/1 hassallidins 18, 20, 21 and 23.

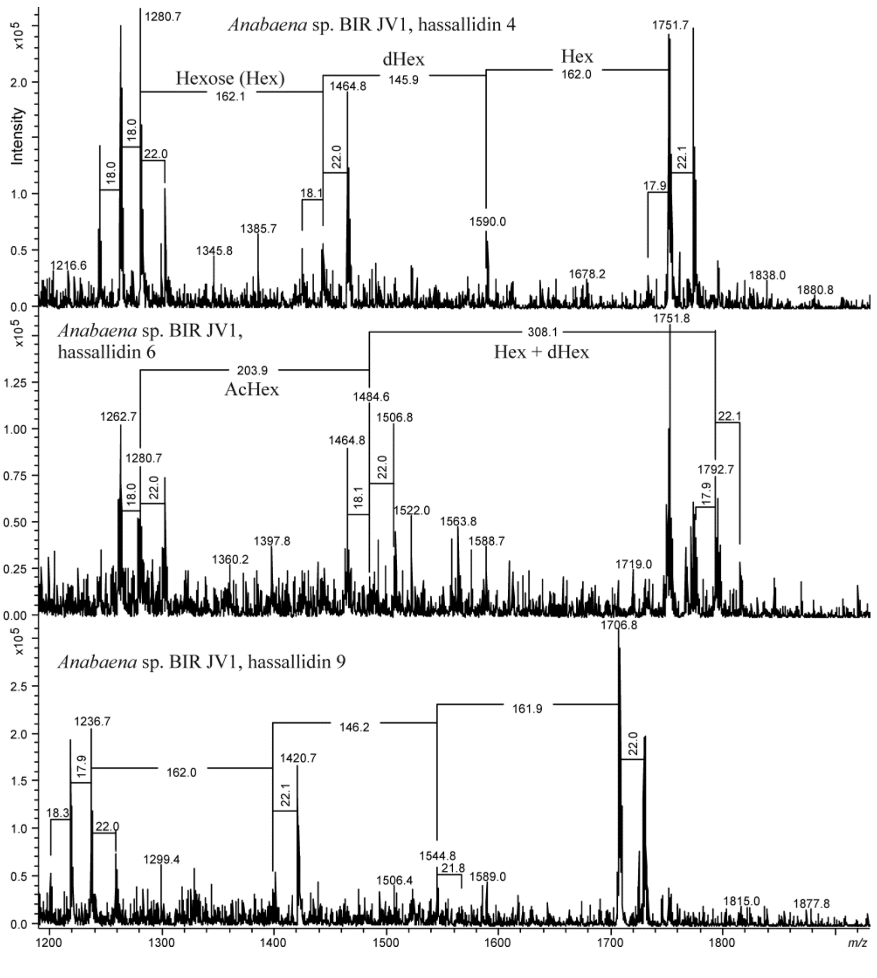

Figure S14. Mass spectra of *Anabaena cylindrica* BIR JV1 hassallidins 4, 6, and 9.

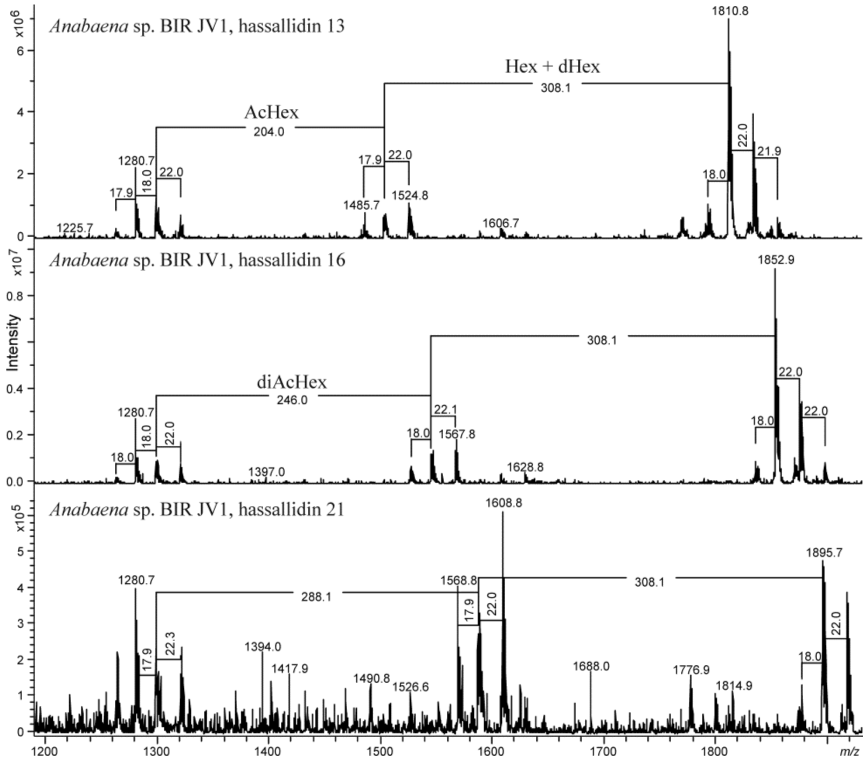

Figure S15. Mass spectra of *Anabaena cylindrica* BIR JV1 hassallidins 13, 16, and 21.

References

1. Moore, R.E.; Patterson, G.M.L; Mynderse, J.S.; Barchi Jr., J.; Norton, T.R.; Furusawa, E.; Furusawa, S. Toxins from cyanophytes belonging to the scytonemataceae. *Pure Appl. Chem.* **1986**, *58*, 263–271.
2. Ishibashi, M.; Moore, R.E.; Patterson, G.M.L. Scytophycins, Cytotoxic and antimycotic agents from the cyanophyte *Scytonema pseudohofmanni*. *J. Org. Chem.* **1986**, *51*, 5300–5306.
3. Carmeli, S.; Moore, R.E.; Patterson, G.M.L. Tolytoxin and new scytophycins from three species of Scytonema. *J. Nat. Prod.* **1990**, *53*, 1533–1542.
4. Nakao, Y.; Yoshida, W.Y.; Szabo, C.M.; Baker, B.J.; Scheuer, P.J. More Peptides and Other Diverse Constituents of the Marine Mollusk *Philinopsis speciosa*. *J. Org Chem.* **1998**, *63*, 3272–3280.
5. Tomsickova, J.; Ondrej, M.; Cerny, J.; Hrouzek, P.; Kopecky, J. Analysis and detection of scytophycin variants by HPLC-ESI-MS. *Chem. Nat. Compd.* **2014**, *49*, 1170–1171.

© 2015 by the authors; licensee MDPI, Basel, Switzerland. This article is an open access article distributed under the terms and conditions of the Creative Commons Attribution license (http://creativecommons.org/licenses/by/4.0/).
